# PAD4-DB: A Curated Structure–Activity Resource Reveals Hub-Organized Activity Cliffs and Scaffold-Dependent SAR Ruggedness in PAD4 Inhibitors

**DOI:** 10.3390/ijms27188302

**Published:** 2026-09-17

**Authors:** Nidhal Tarhouni, Ahmed Bayoudh, Amira Mahfoudhi, Bilel Hadrich, Karim Kriaa, Imen Kallel

**Affiliations:** 1 Laboratory of Enzyme Engineering and Microbiology, Engineering National School of Sfax (ENIS), University of Sfax, P.O. Box 1173, Sfax 3038, Tunisia; nidhal1t@gmail.com (N.T.); ahmed.bayoudh@fss.usf.tn (A.B.); 2 Laboratory of Biochemistry and Enzymatic Engineering of Lipases, Engineering National School of Sfax (ENIS), University of Sfax, Sfax 3038, Tunisia; amira.mahfoudhi95@gmail.com; 3 Department of Chemical Engineering, College of Engineering, Imam Mohammad Ibn Saud Islamic University (IMSIU), Riyadh 11432, Saudi Arabia; kskriaa@imamu.edu.sa; 4 Research Laboratory of Environmental Toxicology-Microbiology and Health (LR17ES06), Faculty of Sciences, University of Sfax, Sfax 3038, Tunisia; kallel1imen@yahoo.fr

**Keywords:** PAD4, PADI4, protein arginine deiminase, activity cliffs, matched molecular pairs, structure–activity relationship, cheminformatics, database

## Abstract

Peptidylarginine deiminase 4 (PAD4) is an increasingly prominent therapeutic target in oncology, inflammatory disease, and neutrophil extracellular trap (NET)-associated pathologies, yet public bioactivity data for PAD4 inhibitors remain fragmented across multiple repositories with substantial redundancy and inconsistent annotation. Here, we present PAD4-DB, a curated structure–activity relationship resource integrating 3093 unique inhibitors (consensus pIC_50_ range 2.00–8.52; median 6.84) from PubChem, ChEMBL, and BindingDB through a reproducible pipeline encompassing structure standardization, activity normalization, source-independence assessment, and deduplication. Quantitative analysis of 358,416 compound pairs with Tanimoto similarity ≥0.6 indicates that the sampled PAD4 SAR landscape is predominantly smooth: only 94 pairs meeting the stringent activity-cliff criterion (Tanimoto ≥ 0.8; |ΔpIC_50_| ≥ 2.0) were identified, representing 0.026% of all related pairs and 0.78% of the 12,071 cliff-candidate pairs. Of these, 80 (85.1%) received structural support from matched molecular pair (MMP) analysis. Severe cliffs were non-randomly distributed, with four hub compounds collectively accounting for 53.2% of all severe cliff pairs, while 96.8% of multi-member scaffold series remained completely smooth. Provenance analysis further showed that 82.9% of the dataset was classified as pipeline-dependent under the provenance-scoring framework across repositories rather than independent source measurements, underscoring the importance of provenance-aware confidence weighting in downstream modeling. PAD4-DB therefore provides a reproducible foundation for PAD4 inhibitor discovery and a curated benchmark for evaluating similarity-based and machine-learning approaches in a chemically structured SAR landscape containing rare but highly concentrated activity cliffs.

## 1. Introduction

Protein arginine deiminase 4 (PAD4, encoded by the *PADI4* gene; UniProt Q9UM07) is a calcium-dependent enzyme that converts peptidyl-arginine into peptidyl-citrulline. This post-translational modification functions as a crucial regulator of chromatin decondensation, gene transcription, and the formation of neutrophil extracellular traps (NETs) [1]. Beyond its established role in autoimmune and inflammatory disease, PAD4 is markedly overexpressed in multiple human cancers, where it plays a key role as a transcriptional corepressor of p53 through histone citrullination and contributes to oncogenic transcriptional programs, establishing PAD4 as an emerging therapeutic target in oncology [2,3,4]. Citrullinated proteins serve as the principal antigens targeted by disease-defining anti-citrullinated protein antibodies [5]. Furthermore, *PADI4* Polymorphisms are linked to rheumatoid arthritis susceptibility, and pharmacological inhibition suppresses disease severity in preclinical models [5,6,7]. Beyond joint disease, dysregulated citrullination drives NET-mediated thrombosis, sepsis, neurodegeneration, and tumor progression, the latter through NET-dependent promotion of metastatic dissemination and suppression of antitumor immunity [5,6,7,8]. Mechanistically, a narrow, calcium-gated active site anchored by the Cys645–His471–Asp473 catalytic triad exhibits extreme sensitivity to ligand geometry [9,10], a constraint that leaves an imprint on inhibitor structure–activity relationships.

Consequently, PAD4 has attracted sustained drug-discovery effort, reviewed comprehensively elsewhere [11], across several chemotype generations as follows: covalent haloacetamidine probes (F-amidine, Cl-amidine, and BB-Cl-amidine) [12,13,14,15]; ortho-substituted amidine analogs with improved isozyme selectivity [16]; reversible, PAD4-selective benzimidazoles (GSK199, GSK484) that established selective enzymatic inhibition as sufficient to disrupt NET formation [17]; indazole- and naphthalene/quinoline-based scaffolds optimized for potency and metabolic stability [18,19]; and most recently allosteric pan-PAD inhibitors acting through a calcium-dependent binding site [20]. Clinical translation is actively advancing: JBI-589 was developed to suppress tumor-associated PAD4-mediated neutrophil migration, thereby limiting metastatic progression and enhancing the efficacy of immune-checkpoint blockade [21]. Additionally, the oncology-directed inhibitor BMS-P5 blocks NET formation and delays progression of multiple myeloma in preclinical models [22]; an orally available inhibitor alleviates arthritis in preclinical models [23]. Furthermore, patent analyses document twenty-eight industrial campaigns from ten companies over the past decade, spanning both autoimmune and oncology indications [24]. Supplementary compounds, including TDFA studied for acute lung injury [25] and GSK484, which has also been investigated as a radiosensitizer in cancer therapy [26], populate the public bioactivity record that PAD4-DB sets out to organize.

Despite this sustained activity, the public PAD4 record remains fragmented. Measurements span heterogeneous assay formats, including colorimetric BAEE hydrolysis, fluorescence RFMS, fluorescence-polarization binding, and irreversible-inhibition kinetics, and are reported using incompatible endpoints such as IC_50_, K_i_, Kd, and percent inhibition across PubChem, ChEMBL, and BindingDB. These repositories also re-curate overlapping measurements and therefore do not necessarily represent independent experimental observations. Raw records further contain salt forms, vendor-specific extended-SMILES annotations, and entries lacking usable molecular structures. Together, these issues complicate the following three objectives: (i) construction of a unit-consistent potency variable; (ii) assessment of cross-source concordance while accounting for shared provenance; and (iii) systematic characterization of the activity-cliff landscape, where structurally similar compounds exhibit unexpectedly large potency differences and similarity-based predictive models often perform poorly [27].

Activity cliffs, structurally similar compound pairs with unexpectedly large potency differences, identify regions where the similarity principle breaks down and where similarity-based models underperform most severely [28,29]. Systematic benchmarking across thirty targets showed that molecular machine-learning methods generally perform substantially worse on activity-cliff compounds [27], and even interpretation methods can provide unreliable explanations in these regions on precisely the compounds medicinal chemists most need to interpret [30]. Whether activity cliffs are broadly distributed or concentrated around a small number of hub compounds is therefore of direct practical importance, yet this organization has not been characterized for PAD4. Scaffold-distribution metrics are an equally important descriptor of chemotype structure [31,32], and the recognition that structure–activity landscapes are heterogeneous, interleaving smooth regions with abrupt discontinuities, has reframed SAR analysis as navigating terrain rather than fitting a global trend [33,34]. Given the growing interest in PAD4 as a therapeutic target in oncology [2,4,8], defining where the sampled structure–activity landscape is predominantly smooth and where it becomes locally rugged is directly relevant to prioritizing analog optimization in anticancer drug-discovery programs, in addition to autoimmune applications.

In this study, we develop PAD4-DB, a systematically curated structure–activity resource for PAD4, together with a provenance-aware analysis of its structure–activity landscape. We first harmonize heterogeneous bioactivity records into a standardized dataset of 3093 inhibitors with comparable pIC_50_ measurements, providing a consistent basis for quantitative SAR analysis. We then determine how much apparent cross-source concordance can be attributed to shared repository provenance rather than independent experimental evidence among public repositories. Using the resulting provenance-aware dataset, we characterize the organization of PAD4 chemical space and determine whether activity cliffs are broadly distributed or concentrated around specific scaffold-defined regions and hub compounds. Finally, we test the stability of these landscape features across fingerprint representations and use matched molecular pair (MMP) analysis to provide an orthogonal assessment of the structural transformations associated with pronounced potency discontinuities.

## 2. Results

### 2.1. PAD4-DB Curation and Composition

We constructed PAD4-DB by integrating data from 57 confirmatory and 11 literature-derived PubChem bioassays (Layer A/C), 26 secondary PubChem assays (Layer D/E) [35], three high-throughput screening (HTS) campaigns (AIDs 463073, 485272, and 488796), the ChEMBL assay CHEMBL6111 [36], and the BindingDB record for UniProt Q9UM07 (Section 4) [37,38]. A stringent curation pipeline ingested 341,282 measurement rows, standardized molecular structures using RDKit [39], normalized activity values, aggregated technical replicates, and deduplicated entries by InChIKey within each source and endpoint type. Structure standardization succeeded for 341,276 of the 341,282 rows (99.998%), with no parse or sanitization failures after removing BindingDB Daylight extended-SMILES annotations and salts. Activity normalization produced 338,021 valid endpoint records (99.0%); notably, HTS percent-inhibition measurements (*n* = 330,136) were intercepted before unit conversion to prevent erroneous conversion into nanomolar potency values.

**Figure 1:**
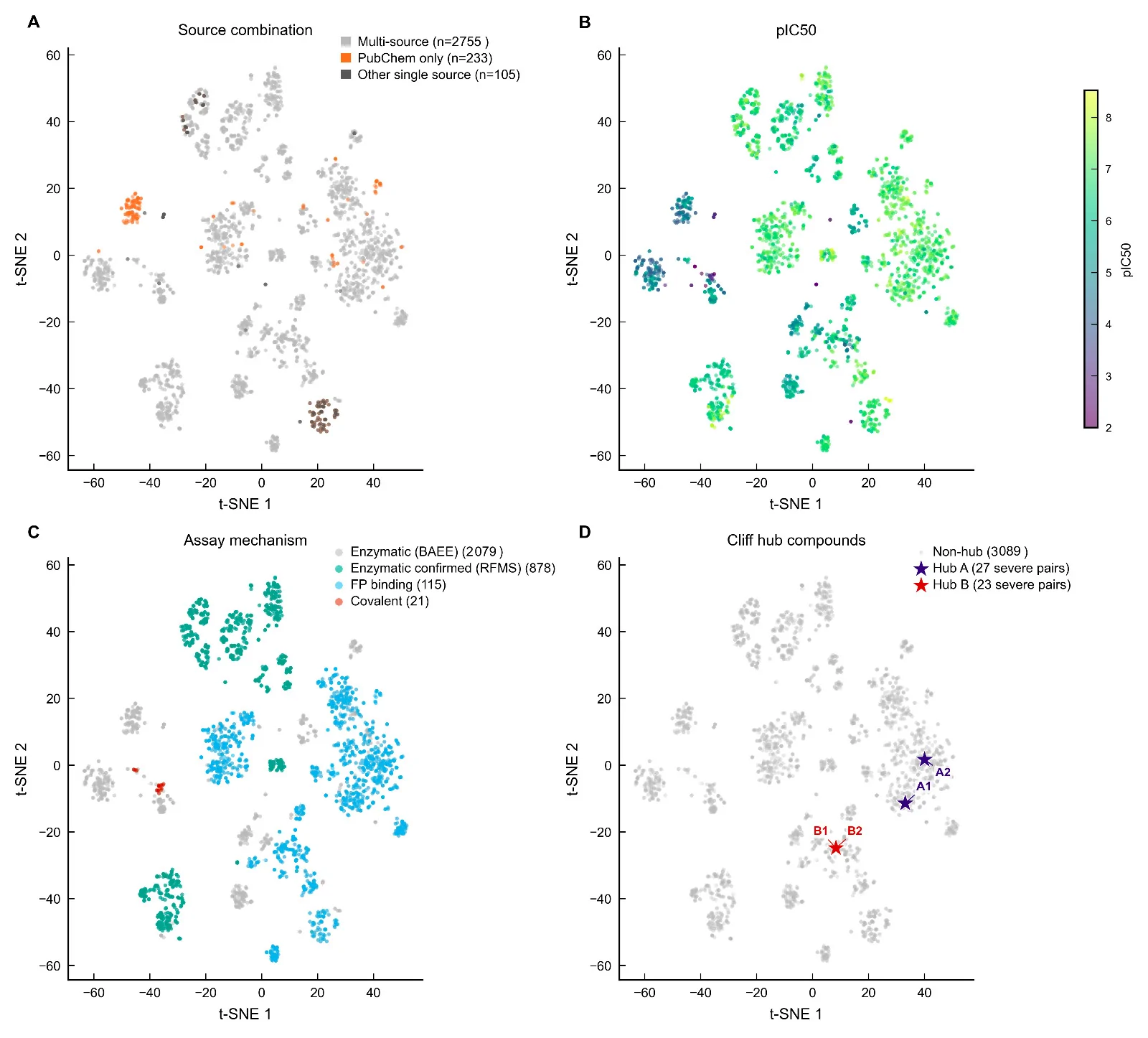
Chemical-space landscape of the PAD4-DB potency set. Two-dimensional t-SNE embedding of ECFP4 fingerprints (Morgan radius 2, 2048 bits; RDKit 2025.09.5) for all 3093 inhibitors. (**A**) Source combination: multi-source (*n* = 2755), PubChem-only (*n* = 233), and other single-source (*n* = 105; BindingDB- or ChEMBL-only) compounds occupy distinguishable regions of chemical space. (**B**) Consensus pIC_50_ (viridis scale, range 2.00–8.52). (**C**) Assay mechanism: enzymatic BAEE (*n* = 2079), RFMS-confirmed enzymatic (n = 878), fluorescence-polarization binding (*n* = 115), and covalent (*n* = 21) measurements. (**D**) The four severe-cliff hub compounds Class A (A1, A2; 27 severe cliff pairs) and Class B (B1, B2; 23 severe cliff pairs) against the non-hub background (*n* = 3089). t-SNE perplexity = 30, 1000 iterations, random seed fixed; embedding used for visualization only.

The resulting resource comprises the following two complementary layers: a dose–response potency dataset containing 3093 unique inhibitors with consensus pIC_50_ values and a parallel HTS dataset comprising 327,336 unique compounds with single-concentration percent-inhibition measurements. Together, these data define the chemical space, potency landscape, and assay composition of PAD4-DB (Figure 1A–C). Two ChEMBL entries with qualifying IC_50_ measurements but no deposited chemical structures were excluded. The final dose–response resource therefore comprises 3093 structure-resolved compounds. A complete per-compound field listing potency, source and assay-mechanism annotations, source-independence score, scaffold assignment, and activity-cliff/hub membership is provided as Supplementary Table S5, together with an accompanying field dictionary.

The curated inhibitors span a pIC_50_ range of 2.00–8.52 (mean 6.55, median 6.84, and SD 0.99) and exhibit a bimodal potency distribution, with a dominant peak near pIC_50_ 7.0 and a lower-potency shoulder around pIC_50_ 5.0–6.0 driven primarily by patent-exclusive screening hits.

The chemical space is dominated by multi-source compounds (*n* = 2755), whereas PubChem-only (*n* = 233) and other single-source compounds from BindingDB or ChEMBL (*n* = 105) occupy more peripheral regions of the embedding (Figure 1A). Assay mechanisms are similarly heterogeneous, with enzymatic BAEE assays (*n* = 2079) and RFMS-confirmed enzymatic assays (*n* = 878) accounting for most measurements, alongside smaller groups of fluorescence-polarization binding (*n* = 115) and covalent assays (*n* = 21) (Figure 1C). Coloring the embedding by consensus pIC_50_ shows that potent and weak inhibitors are broadly distributed across chemical space rather than confined to a single chemotype (Figure 1B). The four severe activity-cliff hub compounds (A1–A2 and B1–B2) occupy distinct regions of the embedding (Figure 1D), highlighting localized regions in which structurally similar compounds exhibit large potency differences. Together, these observations provide an initial view of a broadly heterogeneous PAD4 chemical space containing localized regions of pronounced SAR discontinuity.

### 2.2. Source Overlap and Independence

**Figure 2:**
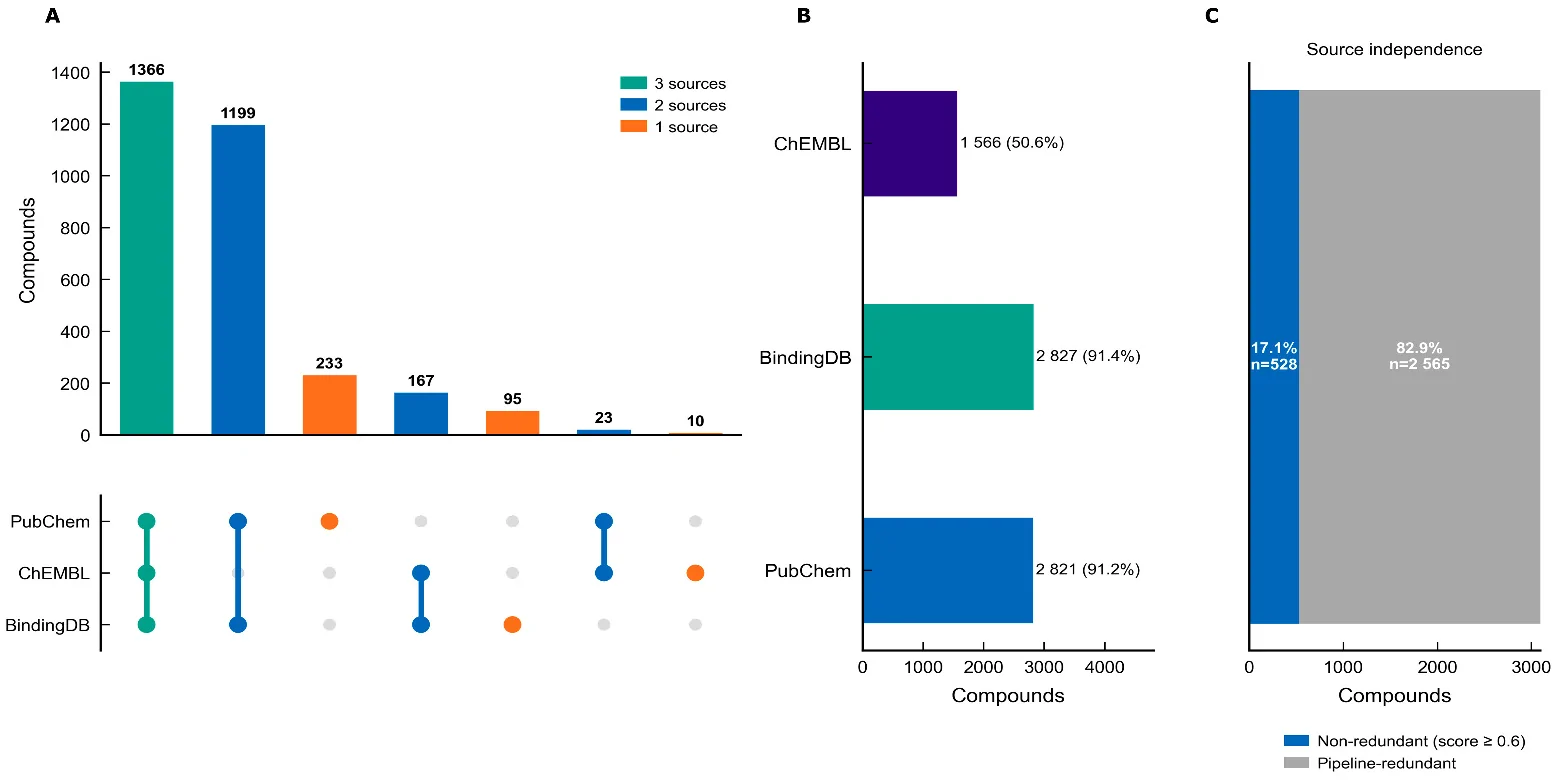
Source composition and independence. (**A**) UpSet plot [40] of source-combination membership across PubChem, ChEMBL, and BindingDB. (**B**) Per-source compound coverage. (**C**) Source-independence partition: 528 compounds classified as non-redundant (17.1%) versus 2565 compounds classified as pipeline-dependent under the heuristic framework (82.9%). Independence scores were derived from source-combination re-curation links (Methods); a threshold ≥ 0.6 defines the non-redundant category.

Although 2755 of the 3093 compounds (89.1%) are represented in more than one public repository, this extensive overlap primarily reflects cross-curation rather than independent experimental replication. An UpSet analysis of PubChem, ChEMBL, and BindingDB shows that the two dominant source combinations—BindingDB + ChEMBL + PubChem (*n* = 1366) and BindingDB + PubChem (*n* = 1199)—together account for 2565 compounds (82.9%) (Figure 2A). In contrast, single-source records are relatively uncommon, comprising 233 PubChem-only compounds, 95 BindingDB-only compounds, and 10 ChEMBL-only compounds.

Per-source coverage further illustrates this redundancy (Figure 2B). PubChem and BindingDB each cover more than 90% of the curated inhibitor set (2821 and 2827 compounds, respectively), whereas ChEMBL contains 1566 compounds (50.6%). Because most compounds are deposited in multiple repositories, these coverage values are not independent and therefore cannot be interpreted as evidence of experimental replication. Instead, they largely reflect re-curation of the same underlying bioactivity studies across public databases.

To distinguish repository overlap from provenance independence, we assigned each compound a source-independence score that penalizes known re-curation relationships among the three databases (Methods). Single-source compounds receive the highest confidence score (1.0), whereas compounds represented across all three repositories receive the lowest score because their records are more likely to derive from shared underlying experimental campaigns. Intermediate scores reflect the empirically observed hierarchy of cross-curation between repositories.

Applying a threshold of ≥0.6 identified 528 compounds (17.1%) as non-redundant, whereas the remaining 2565 compounds (82.9%) were classified as pipeline-dependent through repository re-curation (Figure 2C). This classification was robust to threshold selection: at a stricter threshold of ≥0.7, 361 compounds (11.7%) remained non-redundant, whereas at a more permissive threshold of ≥0.5, 1727 compounds (55.8%) qualified. Sensitivity analyses further supported the stability of the ranking: across 1000 random ±20% perturbations of the weighting scheme, compound rankings remained highly correlated (Spearman ρ = 0.99; minimum ρ = 0.93), while an alternative inverse-source-count weighting produced a similarly high correlation (ρ = 0.98). Accordingly, we interpret the source-independence score as an ordinal measure of provenance confidence rather than a precise quantitative estimate of experimental independence. The high apparent cross-source concordance cannot be interpreted as evidence of independent experimental validation and is substantially influenced by shared data provenance, underscoring the importance of provenance-aware confidence metrics when integrating public bioactivity resources.

### 2.3. Assay-Dependent Potency Structure

**Figure 3:**
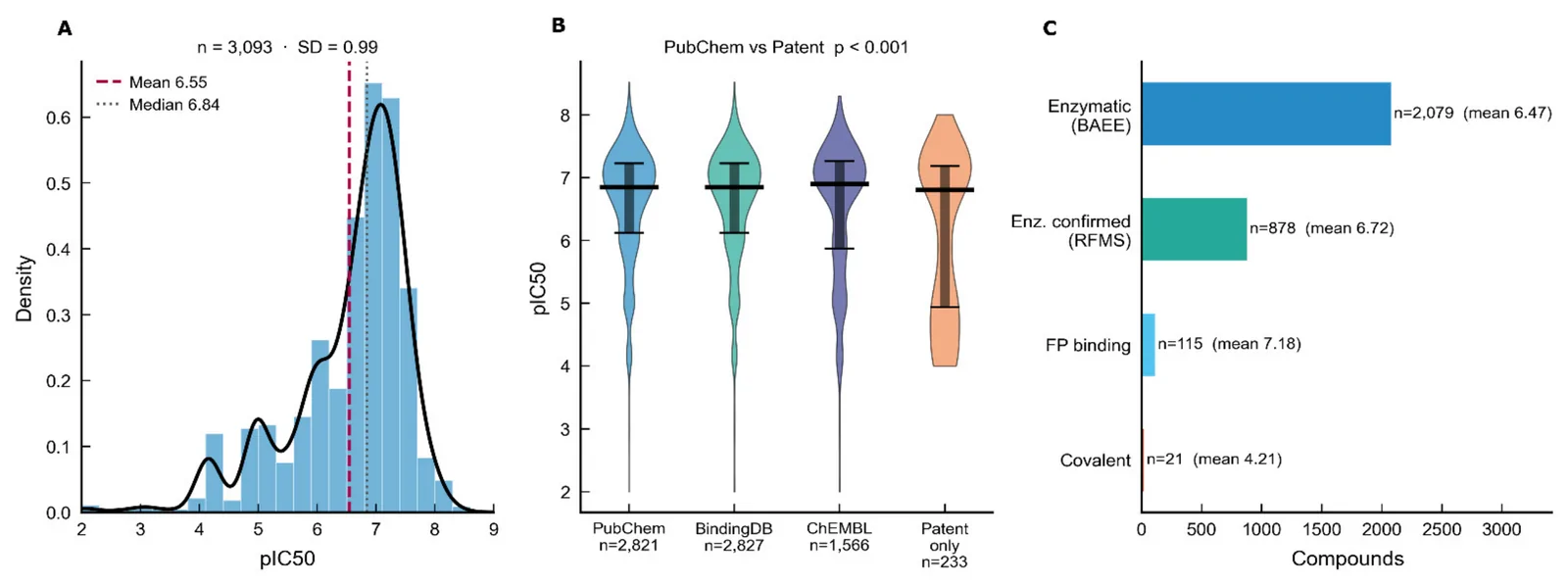
Potency landscape. (**A**) Consensus pIC_50_ distribution (histogram + KDE; mean 6.55, median 6.84, SD 0.99; n = 3093). (**B**) pIC_50_ distributions by source and patent-exclusive status (violin plots; medians marked). The difference between published and patent-exclusive compounds is significant (Mann–Whitney U, two-sided; *p* = 1.3 × 10^−5^, rank-biserial r = 0.17). (**C**) Compound counts and mean pIC_50_ values for each assay-mechanism class. Each compound is assigned to a single primary assay-mechanism class; n values sum to 3093.

To assess whether database provenance was associated with reported potency, we compared consensus pIC_50_ values across the three repositories. The source-associated potency distributions showed no systematic difference in central tendency (PubChem mean pIC_50_ 6.62; BindingDB 6.59; ChEMBL 6.50; and Kruskal–Wallis H = 0.25, *p* = 0.88; Table 1). Because compounds can occur in multiple repositories, this comparison was interpreted as descriptive rather than as an independent-group inference. Nevertheless, the overall potency distribution showed a dominant peak near pIC_50_ 7.0 and a lower-potency shoulder around 5.0–6.0 (Figure 3A). Source origin therefore appeared to affect distributional shape more than central tendency (Figure 3B). Patent-exclusive compounds (*n* = 233), corresponding to the PubChem-only single-source records in our curation, had a median pIC_50_ nearly identical to that of non-patent-exclusive compounds (Δmedian = 0.04), but exhibited a heavier low-potency tail and consequently a lower mean pIC_50_ (6.13 versus 6.53; Mann–Whitney U, two-sided, *p* = 1.3 × 10^−5^, and rank-biserial r = 0.17). Potency also correlated moderately with molecular size, measured by molecular weight (Spearman ρ = 0.54) and heavy-atom count (ρ = 0.57; both *p* < 10^−220^), identifying molecular size as an important covariate for subsequent ligand-efficiency analyses. Electrophilic warheads were detected in 107 of 3093 compounds (3.5%), predominantly chloroacetamidine (*n* = 66), fluoroacetamidine (*n* = 17), haloacetyl (*n* = 11), and other minor electrophilic classes (n = 13). Among the 99 unique inhibitors participating in severe cliffs, only one was patent-exclusive (Fisher’s exact test, *p* = 0.006; odds ratio = 0.12), indicating significant underrepresentation of patent-exclusive compounds among severe-cliff compounds.

In contrast, potency differed substantially across primary assay-mechanism classes (Figure 3C). Fluorescence-polarization binding assays showed the highest mean potency (pIC_50_ 7.18, *n* = 115), followed by RFMS-confirmed enzymatic measurements (pIC_50_ 6.72, *n* = 878). The majority of measurements were generated by enzymatic BAEE colorimetric assays, which showed an intermediate mean potency (pIC_50_ 6.47, *n* = 2079). Covalent-mechanism measurements exhibited the lowest mean potency (pIC_50_ 4.21, *n* = 21). This separation is consistent with differences in assay format and inhibitor mechanism, including the sensitivity of single-timepoint IC_50_ measurements to time-dependent inhibition by irreversible inhibitors [41]. Together, these results indicate that we did not detect a systematic difference in consensus pIC_50_ distributions among the three source databases, whereas assay-mechanism-related variance remained evident, downstream analyses account for this by reporting assay-mechanism annotations alongside potency values and confirming that severe activity cliffs are not enriched in any single assay-mechanism class (Section 2.8).

### 2.4. Reference Inhibitor Validation

We audited 14 curated PAD4 reference inhibitors (Table 2). The following seven of these are present in our dataset with consensus pIC_50_ values that closely match the literature (mean |ΔpIC_50_| < 0.15): GSK484 (7.049), BMS-P5 (7.009), TDFA (5.638), Streptonigrin (5.602), Cl-amidine (5.219), F-amidine (4.571), and JBI-589 (6.000). Three compounds are present in the raw data but were correctly excluded from the final dose–response potency set because they lack a primary IC_50_ endpoint (o-F-amidine, kinact/KI only; Amodiaquine, HTS only; and BB-Cl-amidine, covalent kinetics only). Three reference inhibitors are absent from all source databases (GSK199, Pyroxamide, PAD-PF1), reflecting public curation gaps rather than pipeline errors, and one compound (AFM-30a) was correctly excluded as PAD2-selective. GSK484’s salt-form discrepancy was also resolved: after standardization, the pipeline assigned the free-base InChIKey (BDYDINKSILYBOL-WMZHIEFXSA-N).

### 2.5. Scaffold Architecture and SAR Ruggedness

**Figure 4:**
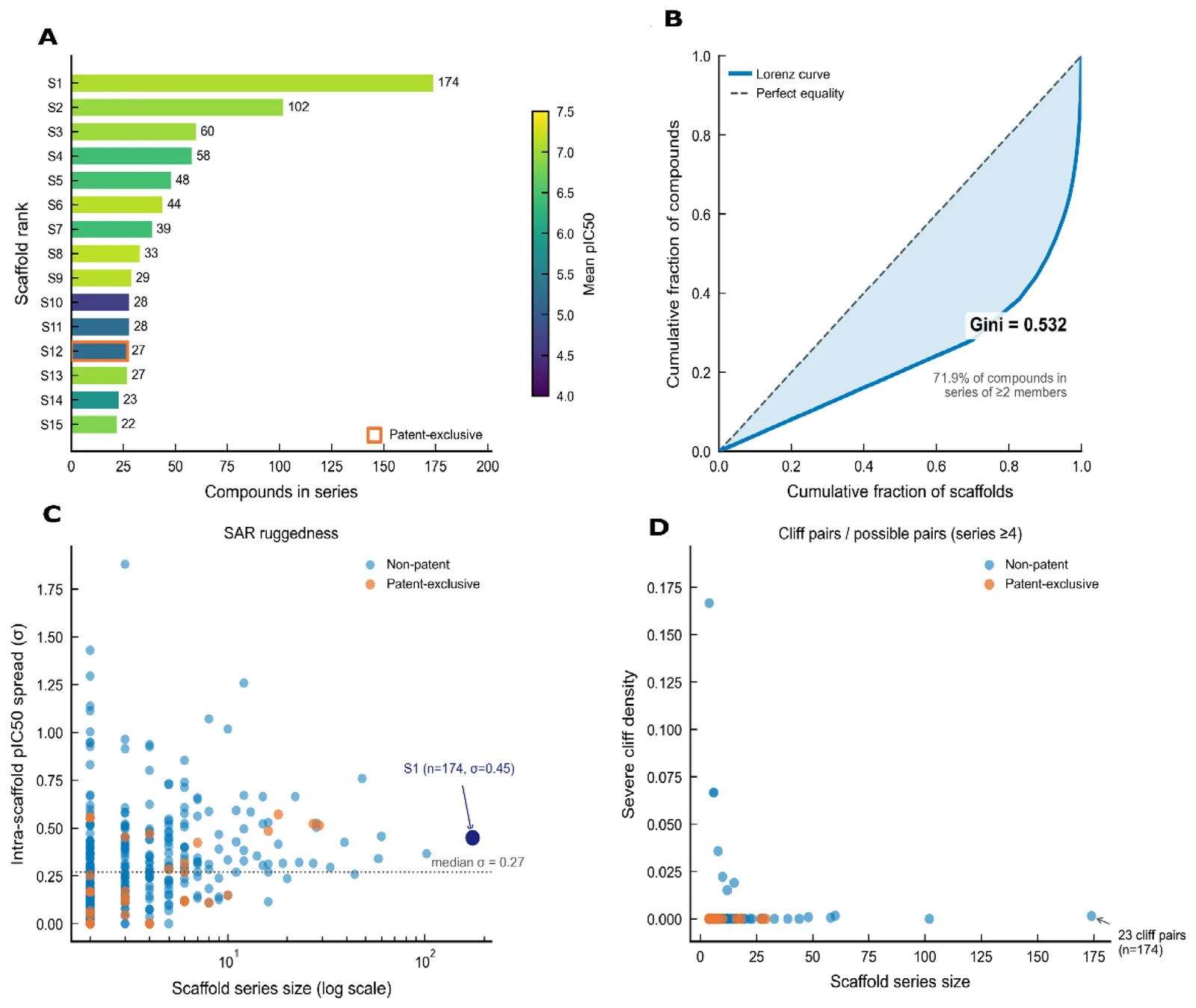
Scaffold landscape and SAR ruggedness. (**A**) Top-15 Murcko scaffold series by compound count, colored by mean pIC_50_; patent-exclusive scaffolds are outlined in orange. (**B**) Lorenz curve of the scaffold-size distribution (Gini = 0.532); 71.9% of compounds belong to series with ≥2 members. (**C**) Series size (log scale) versus intra-scaffold pIC_50_ spread (σ); the median σ = 0.27 is marked with a dotted line, and the largest series (S1) is labeled. (**D**) Severe-cliff density for series with ≥2 members; the vast majority harbor zero cliff pairs, with the largest series (S1) containing 23 severe cliff pairs. σ = within-series SD of consensus pIC_50_. Generic Bemis–Murcko frameworks were generated with RDKit 2025.09.5.

The 3093 inhibitors map to 1244 unique Bemis–Murcko scaffolds, comprising 375 multi-member series and 869 singletons. This distribution is highly uneven and characteristic of series-dominated bioactive chemical space [42] and is reflected in a pronounced concentration of chemotypes (Gini = 0.532). The largest series, an azaindole–benzimidazole framework, contains 174 compounds, and 71.9% of all inhibitors belong to series with at least two members (Figure 4A,B). Notably, 103 of the 233 patent-exclusive compounds occupy scaffolds not otherwise represented in the non-patent-exclusive set. These unique chemotypes occur at a similar series density to the non-patent-exclusive compounds (mean 2.5 members) but exhibit moderately lower mean potency (pIC_50_ 6.13 versus 6.53).

We observed heterogeneous intra-scaffold potency spread, providing evidence of scaffold-dependent SAR ruggedness (Figure 4C). The median within-series spread is σ = 0.27 log units; however, several individual series have σ > 1.0, and the dominant 174-member series has σ = 0.45 and spans a 2.66 log-unit pIC_50_ range. Despite this variation, ruggedness remains the exception rather than the rule. Only 12 of 375 multi-member series (3.2%) contain any within-scaffold severe activity cliffs, leaving 96.8% completely smooth (Figure 4D; Supplementary Figures S1 and S4, Supplementary Table S1). Although ruggedness moderately correlates with series size (Spearman ρ = 0.36, *p* = 5 × 10^−13^), size alone is insufficient to predict it; the second-largest series (*n* = 102) remains completely smooth (σ = 0.37, zero cliffs) despite being sampled nearly as densely as the largest series. Ruggedness is therefore highly scaffold- and vector-specific.

### 2.6. Activity-Cliff Landscape

Across 358,416 structurally related pairs (Tanimoto ≥ 0.6), including 12,071 cliff-candidate pairs with Tanimoto ≥ 0.8, the SAS map (Supplementary Figure S3) is overwhelmingly dominated by smooth SAR: 96.06% are non-descript, 3.34% continuous, 0.57% discontinuous, and only 0.026% (94 pairs) constitute severe activity cliffs (Tanimoto ≥ 0.8, |ΔpIC_50_| ≥ 2.0). Against an unrestricted permuted-potency null model (pIC_50_ shuffled across 2620 compounds within the Tanimoto ≥ 0.8 subgraph; 10,000 permutations), these 94 cliffs represent a 13-fold depletion relative to the null expectation of 1225 ± 100 cliffs (*p* < 0.001). Notably, when permutations are constrained within Murcko scaffolds, this depletion is abolished (null expectation, 101 ± 13 cliffs; 1.1-fold, *p* = 0.32; Supplementary Figure S5). Together, these results indicate that the apparent global smoothness is largely attributable to potency homogeneity within sampled scaffold series rather than an intrinsic property of the broader chemical space.

The |ΔpIC_50_| ≥ 2.0 threshold was selected as a stringent criterion for identifying large potency discontinuities, consistent with activity-cliff definitions that combine high structural similarity with substantial potency differences [28,30]. Because cross-source agreement in this dataset is affected by shared pipeline re-curation, it cannot be used as a direct estimate of independent assay noise. Nonetheless, the results are robust to threshold choice: the four highest-degree hub compounds remain approximately 3.9-fold enriched across |ΔpIC_50_| cut-offs of 1.5, 2.0, and 2.5 (Supplementary Table S2). Applying graded |ΔpIC_50_| thresholds while retaining Tanimoto ≥ 0.8 yields 94 severe (|ΔpIC_50_| ≥ 2.0), 193 moderate (1.5–<2.0), and 580 broad (1.0–<1.5) cliff pairs, involving 99, 209, and 539 compounds, respectively (Table 3). The maximum observed severe-cliff potency difference is 3.045 log units (≈1100-fold), and the mean severe |ΔpIC_50_| is 2.31.

### 2.7. Hub Organization of Severe Cliffs

**Figure 5:**
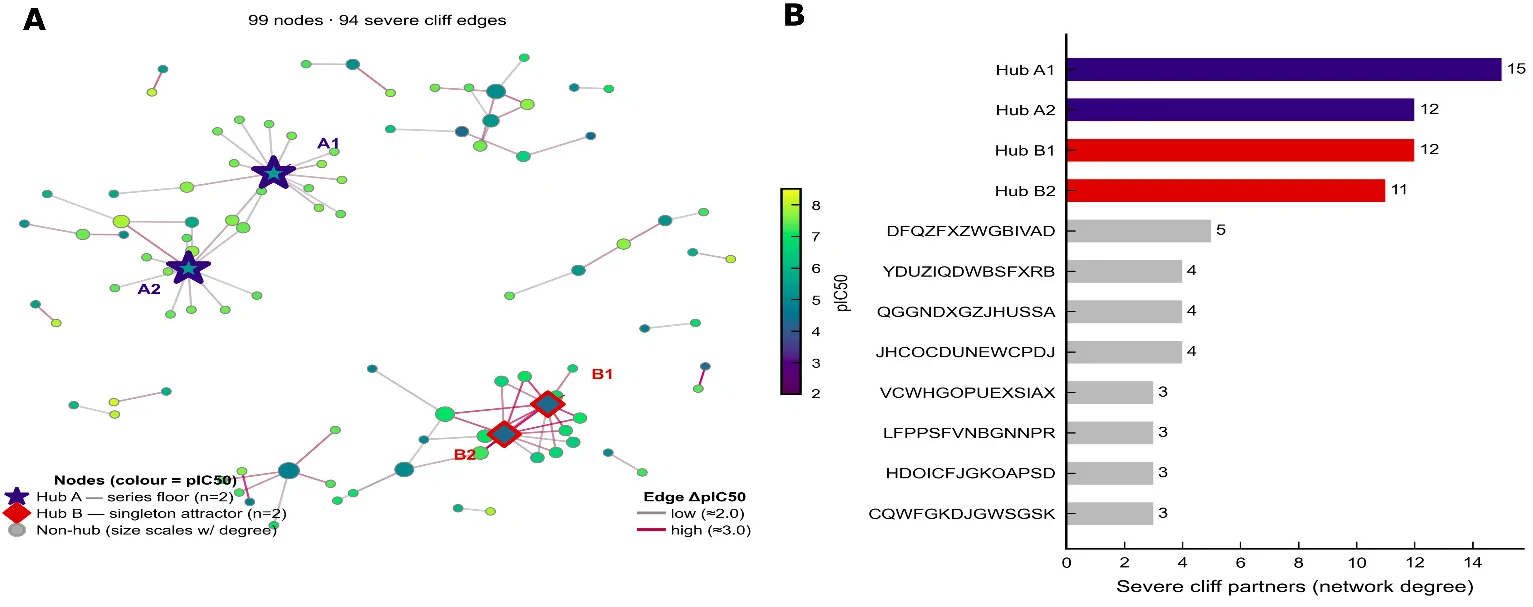
Severe activity-cliff network. (**A**) Ninety-nine compounds (nodes; size ∝ degree; color = pIC_50_ viridis) connected by 94 severe cliff edges (color = |ΔpIC_50_|). Class A hubs (navy stars), Class B hubs (red diamonds). (**B**) Top-12 compounds by degree. Spring layout, fixed seed. Hub enrichment: unrestricted 3.9×, *p* < 0.001; scaffold-constrained z = 2.6, *p* = 0.011; assay-constrained 4.0×, *p* < 0.001.

The severe-cliff network comprises 99 nodes and 94 edges and is dominated by four high-degree hubs that together account for 50 of the 94 pairs (53.2%) (Figure 5 and Table 4). These four hubs are separated from the remainder of the network by a clear degree discontinuity as follows: their degrees (15, 12, 12, and 11) are followed by a fifth-ranked compound with degree 5, corresponding to a 2.20-fold decrease and the largest local degree discontinuity in the ranked network. Sensitivity analysis using alternative top-*K* definitions showed that the concentration of severe-cliff connectivity among highly connected compounds was maintained across *K* = 3–6, supporting the use of the four-compound cutoff as a data-driven cutoff based on the observed degree distribution. This hub concentration significantly exceeds expectations under the scaffold-constrained permutation (53.2% versus 40.1 ± 5.1%; *z* = 2.6, *p* = 0.011), the assay-constrained null model (4.0-fold, *p* < 0.001), and the unrestricted null model (3.9-fold, *p* < 0.001). Thus, the observed hub concentration is unlikely to be explained solely by scaffold composition, assay composition, or cross-assay comparisons.

**Figure 6:**
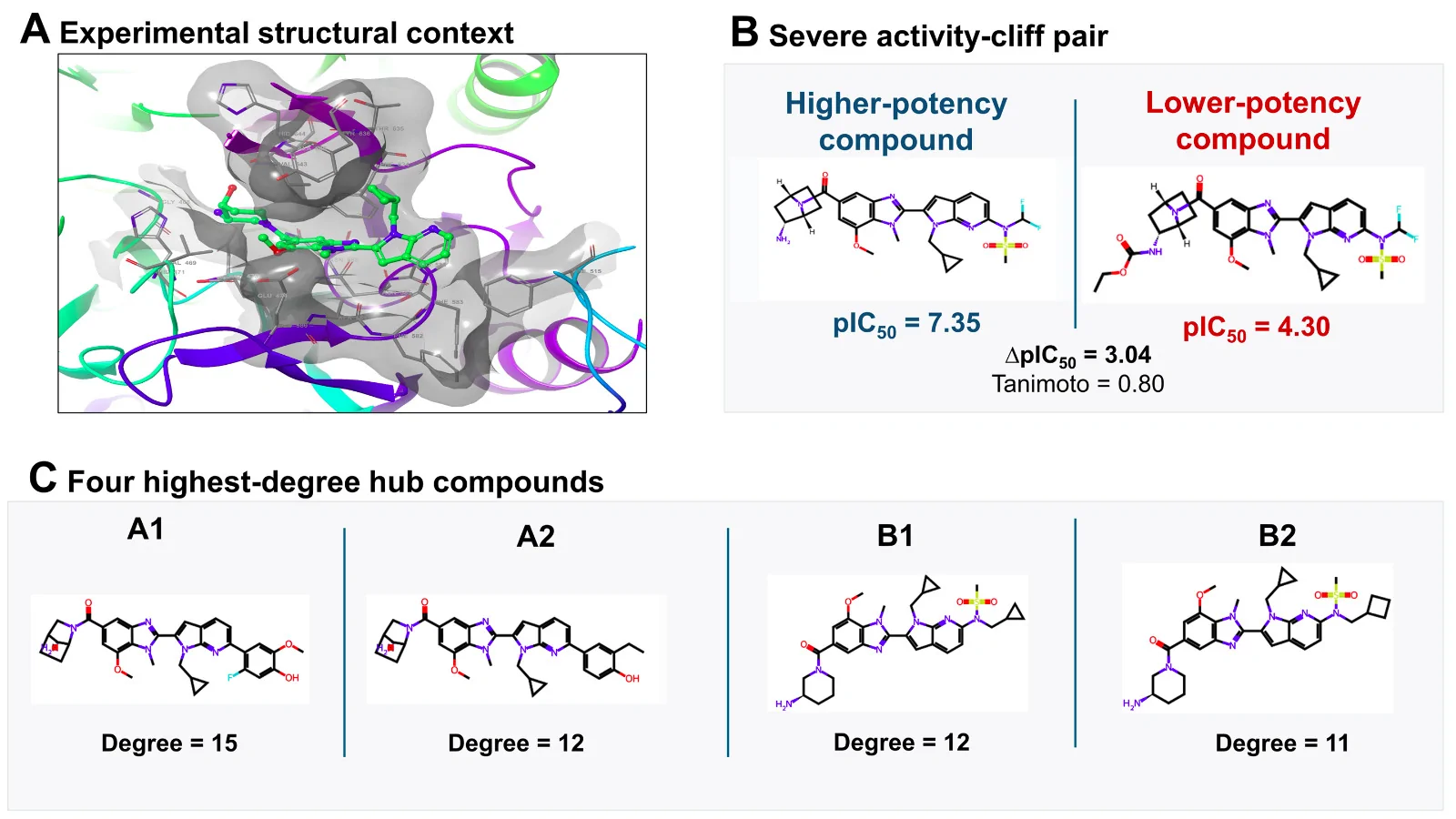
Experimental structural context and network-defined activity-cliff architecture of PAD4 inhibitors. (**A**) Crystal structure of human PAD4 in complex with GSK147 (PDB ID 4 × 8C), providing an experimentally determined structural context for the PAD4 inhibitor-binding environment. (**B**) Representative severe activity-cliff pair identified in PAD4-DB, showing a 3.04-unit difference in consensus pIC_50_ despite high structural similarity (Tanimoto = 0.80). (**C**) Four highest-degree compounds in the severe activity-cliff network, designated A1–B2, with degrees of 15, 12, 12, and 11, respectively. The PDB structure is presented as experimental structural context and was not used to infer or validate individual activity-cliff relationships or hub binding modes.

Figure 6 provides experimental structural context for PAD4 chemical space and illustrates a representative severe activity-cliff pair together with the four highest-degree hub compounds.

Class A—series-embedded, sub-median-potency floors. Hubs A1 (SMADULGDNOCLOP-GISFHXKWSA-N; pIC_50_ 5.39, 15 cliff pairs) and A2 (RAVBZQAQTVGKIV-XBPDSQQVSA-N; pIC_50_ 5.34, 12 cliff pairs) are sub-median-potency members of the dominant 174-member azaindole–benzimidazole series. Operating as within-series potency floors, they generate 27 severe cliff pairs against higher-potency analogs (23 of which are contained strictly within the S1 scaffold, with the remainder crossing to closely related frameworks).

Class B—scaffold-singleton structural attractors. Hubs B1 (UDCDEKJNAMHBFH-HSZRJFAPSA-N) and B2 (DVCKJOQIVOGXEI-XMMPIXPASA-N) both have pIC_50_ 4.30 and differ by a single methylene substitution (cyclobutyl versus cyclopentyl sulfonamide; mutual Tanimoto 0.975). Despite their high similarity, they map to distinct Murcko scaffolds because the differentiating ring forms part of the retained framework. This combination of scaffold uniqueness and high fingerprint similarity is consistent with their role as broad structural attractors, generating 23 severe cliff pairs across multiple chemotypes. Their shared free primary amine raises the possibility of assay-interference or nonspecific-binding effects, which should be tested using orthogonal assays.

Physicochemical analyses identified no descriptors that significantly distinguished hub from non-hub cliff compounds after Benjamini–Hochberg correction (Supplementary Table S3). The low-potency signature is specific to the extreme hubs (degree ≥ 5), which are markedly less potent than other cliff compounds (median pIC_50_ 4.82 versus 7.22; rank-biserial r = 0.83; and FDR-adjusted *p* = 0.019). While the broader top-20 set did not show a statistically significant potency difference—likely due to limited statistical power (*n* = 4 vs. *n* ≈ 95)—the magnitude of the effect in the extreme hubs suggests that hub identity is primarily landscape-positional rather than strictly defined by bulk physicochemical properties (Supplementary Table S3).

### 2.8. MMP Support and Fingerprint Robustness

**Figure 7:**
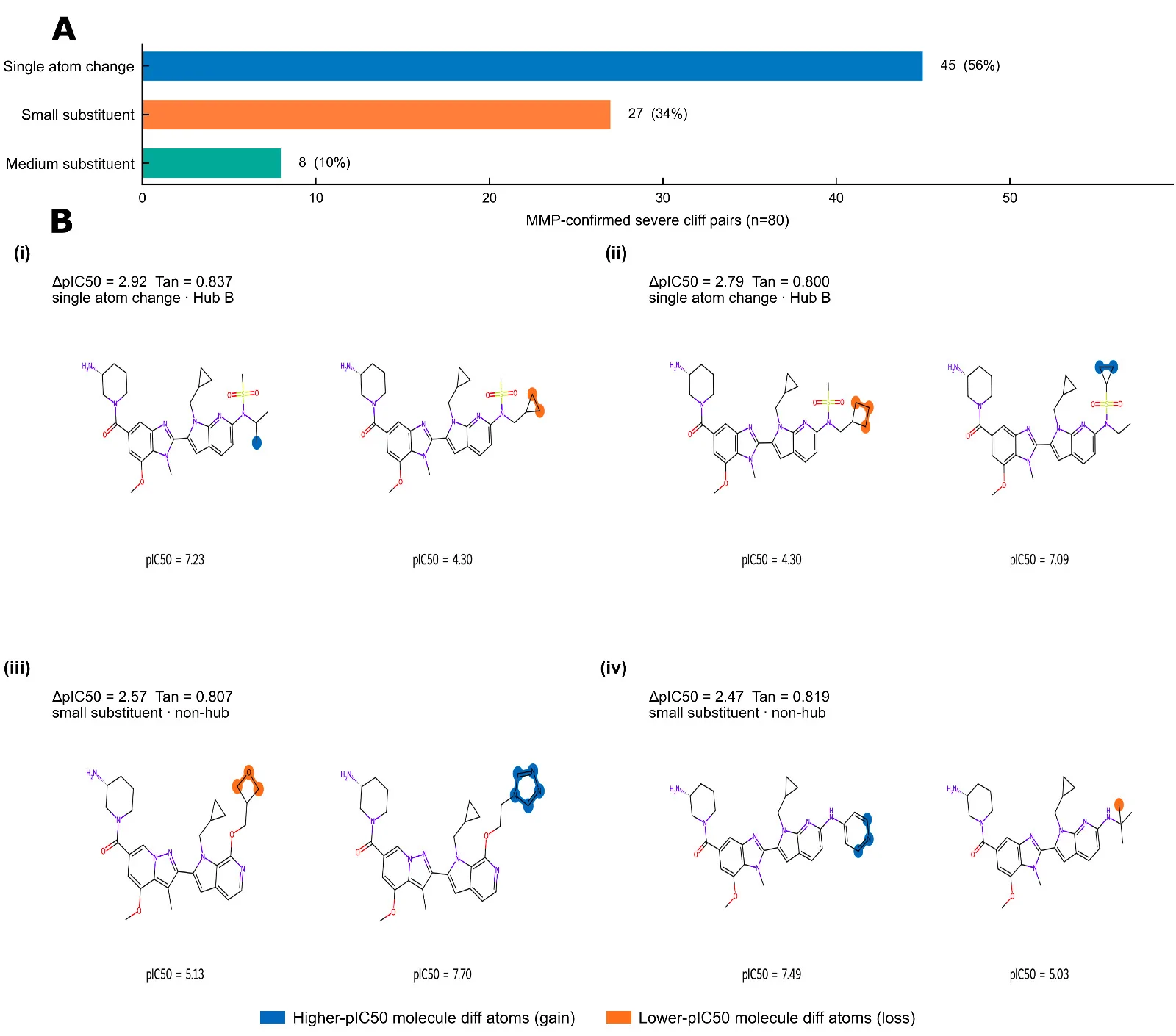
Matched molecular pair analysis of severe cliffs. (**A**) MMP-supported cliff pairs by change type (*n* = 80: 45 single-atom, 27 small-substituent, 8 medium-substituent). (**B**) Four representative cliff pairs; higher-potency difference atoms highlighted in blue (gain), lower-potency in orange (loss). MMP cores from seeded rdFMCS; disconnected fragments excluded.

Matched molecular pair (MMP) analysis provided structural support for 80 of the 94 severe cliff pairs (85.1%) through an explicit shared chemical core (Figure 7A), consisting of 45 single-atom changes (56.3%), 27 small-substituent changes (33.8%), and eight medium-substituent changes (10.0%) (Figure 7B; Supplementary Table S4). We refer to these 80 pairs as MMP-supported severe cliffs and the remaining 14 as fingerprint-only candidate cliffs throughout the remainder of this paper, to distinguish potency discontinuities with an explicit structural correlate from those supported by similarity/potency criteria alone. A sensitivity analysis using ECFP6 (radius 3) revealed that 64 of the 94 severe cliff pairs fall below the Tanimoto 0.80 threshold at the larger radius, as expected for fused-ring systems where an increased radius penalizes shared substructure overlap. Importantly, 51 of these 64 pairs retain MMP support despite falling below the ECFP6 similarity threshold, indicating that the underlying structural transformations remain chemically recognizable despite reduced fingerprint similarity. Hub dominance was preserved across fingerprint representations: the four hubs accounted for 53.2% of severe cliff pairs under ECFP4 and 53.3% under ECFP6. Of the 14 severe cliffs lacking MMP support, 13 also showed reduced ECFP6 similarity and were therefore ECFP4-specific by the chosen threshold; the remaining pair retained high similarity under both fingerprints despite no extractable shared core, suggesting a possible scaffold hop or complex fragment merge rather than a simple localized transformation.

To assess whether pooling mechanistically distinct assays could contribute to artifactual cliffs, we stratified the 94 severe cliff pairs by primary assay mechanism. All 94 cliffs occurred between compounds measured within the same assay-mechanism class; none of the 21 covalent-mechanism compounds participated in any severe cliff pair, and excluding covalent-mechanism compounds entirely left the severe-cliff count unchanged.

Severe-cliff compounds were not enriched in any particular assay-mechanism class (Fisher’s exact test, all *p* > 0.05; Supplementary Figure S2), and 88 of the 94 cliff pairs (94%) shared a common assay identifier, reducing concern that the observed cliffs were driven primarily by cross-assay comparisons. Chemically, the cliffs were highly heterogeneous: 60 pairs (63.8%) involved a heteroatom change, 51 (54.3%) a ring modification, 24 (25.5%) a carbon-only change, 23 (24.5%) halogen substitution, and 13 (13.8%) an aromatic-ring change (categories are non-exclusive). The mean |ΔpIC_50_| remained between 2.2 and 2.5 across all transformation categories (Supplementary Table S4), and the median molecular-weight difference was only 17 Da (55% of pairs < 20 Da). Thus, severe potency discontinuities were associated with relatively small structural changes across a broad range of chemical transformations.

### 2.9. HTS Layer

The 327,336-compound HTS layer provides complementary single-concentration screening context. Among the 1453 dose–response inhibitors that share InChIKeys with HTS-screened compounds, only six (0.4%) were confirmed as HTS actives (≥50% inhibition). The remaining 1447 compounds showed low HTS inhibition (median 4.3%) despite high consensus potency (median pIC_50_ 6.93). This discrepancy may reflect differences in screening concentration, assay format, or experimental context and illustrates the limited comparability of single-concentration HTS measurements with dose–response potency values. Accordingly, the HTS and dose–response layers are best interpreted as complementary measurements rather than as a direct progression from primary screening to validated dose–response activity.

## 3. Discussion

PAD4 has emerged as an important therapeutic target at the intersection of oncology, inflammation, and neutrophil extracellular trap (NET) biology, yet its public medicinal chemistry data remain fragmented across multiple repositories and assay formats. PAD4-DB addresses this gap by transforming heterogeneous public bioactivity records into a standardized, reproducible, and analytically enriched structure–activity relationship (SAR) resource. Beyond data curation, our analysis systematically characterizes the PAD4 activity-cliff landscape, revealing a chemical space in which severe potency discontinuities are rare overall but concentrated around a small number of hub compounds and localized scaffold-defined regions. This organization has direct implications for medicinal chemistry prioritization and for the development and evaluation of predictive models for PAD4 inhibitors in both autoimmune and oncology settings [2,3].

The high apparent cross-source potency agreement is strongly influenced by shared data provenance rather than representing independent experimental validation. At face value, the extensive multi-source overlap observed in PAD4-DB cross-source concordance and 89.1% multi-source fraction could be interpreted as evidence of extensive independent replication. However, applying the proposed source-independence scoring framework, only 17.1% of compounds were classified as non-redundant, indicating that much of the apparent cross-source support arises from shared or re-curated measurements rather than independent experimental observations. Thus, the number of repositories reporting a compound should not be equated with the number of independent experimental confirmations supporting its activity. This distinction is particularly important for medicinal chemistry datasets, where duplicated provenance can inflate the apparent evidential support for individual measurements and complicate assessments of reproducibility. Aggregated bioactivity resources should therefore report provenance-aware measures of source independence alongside conventional multi-source coverage statistics, allowing users to distinguish database redundancy from genuine experimental replication.

The mechanistic interpretations proposed here should be regarded as hypotheses rather than established mechanisms and will require structural and biophysical validation. Potency within the dominant reversible inhibitor chemotypes may depend on a limited number of interactions within the calcium-gated substrate pocket [9,10], such that relatively small structural modifications can disrupt critical interactions and generate disproportionately large potency changes. Class A hubs are consistent with this interpretation, representing compounds positioned along steep local SAR gradients in which analogs differing by a single substituent can span large potency differences within the same chemotype. Such behavior is consistent with heterogeneous structure–activity landscapes in which abrupt activity cliffs coexist with otherwise gradual SAR regions [33].

Class B hubs appear distinct from Class A. These scaffold singletons share a free primary amine and exhibit uniformly low potency while maintaining broad ECFP4 similarity to numerous amine-containing chemotypes. Rather than necessarily representing a shared pharmacophore, they may act as structural attractors that generate cliff relationships across otherwise unrelated scaffold classes [43]. Their identification illustrates the value of combining complementary similarity representations rather than relying exclusively on a single fingerprint [44]. One possible interpretation is therefore that Class A reflects steep local SAR within productive chemotypes, whereas Class B may reflect assay-related, nonspecific, or other context-dependent behavior. These interpretations remain speculative and require orthogonal experimental and structural validation.

Activity-cliff formation appears to require both sufficient local analog density and a sufficiently steep local SAR trajectory. Only 12 of 375 multi-member scaffold series (3.2%) contain any severe activity cliff, indicating that rugged SAR is the exception rather than the rule. Although ruggedness increases with series size (Spearman ρ = 0.36, *p* = 5 × 10^−13^), analog density alone is insufficient to explain this relationship, as the second-largest scaffold class (*n* = 102) remains entirely smooth despite extensive sampling. The location and nature of structural diversification may therefore contribute to whether a scaffold series develops pronounced SAR discontinuities. In particular, substitutions that perturb interactions within the catalytic or calcium-dependent binding environment could plausibly produce larger potency changes than modifications at more permissive positions. This mechanistic interpretation remains hypothetical because no structure-based analysis was performed here. Nevertheless, the observed distribution of ruggedness suggests that SAR difficulty is localized rather than global, allowing medicinal chemistry campaigns to identify scaffold series that warrant denser analog exploration and additional experimental scrutiny.

Comparison with multiple null models indicates that the observed landscape organization is unlikely to be explained by random potency assignment alone. Under the unrestricted null model, severe activity cliffs were globally depleted by approximately 13-fold (*p* < 0.001), whereas this depletion disappeared under scaffold-constrained randomization (1.1-fold, *p* = 0.32). These findings indicate that the apparent global smoothness is largely attributable to the predominance of individually smooth scaffold series rather than to a uniform property of the broader chemical space. In contrast, hub concentration remained significant under both scaffold-constrained (*p* = 0.011) and assay-constrained (*p* < 0.001) null models, indicating that the concentration of severe cliffs around a small number of compounds is not readily explained by scaffold composition or assay composition alone.

Interest in PAD4 inhibition now extends beyond autoimmune disease to oncology, where suppression of PAD4-mediated NET formation has shown promise for limiting metastatic dissemination and enhancing anticancer immune responses [45,46,47,48]. In this context, identifying scaffold series with localized SAR ruggedness may help prioritize analog synthesis, flag regions of elevated optimization uncertainty, and reduce the risk of unexpected potency changes during lead optimization.

The ruggedness map generated here therefore provides a practical framework for prioritization. Within the azaindole–benzimidazole chemotype, transformations associated with severe cliffs should be treated as higher-risk SAR regions because apparently minor structural modifications may produce disproportionately large potency changes. This behavior is exemplified by the hydrolytic conversion of a chloroacetamidine warhead to the corresponding hydroxyacetamidine analog, which reduces potency into the hundred-micromolar range [49]. Conversely, free-amine scaffold singletons resembling the identified Class B hubs warrant confirmation using orthogonal assay formats before being used as anchors for SAR interpretation, particularly because PAD4 activity is influenced by calcium concentration, glycosaminoglycans, and other biological cofactors capable of modifying enzyme activation thresholds [50].

The deposited matched molecular pair (MMP) transformations, which provide structural support for 85.1% of the severe cliffs through an explicitly defined shared core, constitute a PAD4-specific resource for structure-guided SAR analysis [51]. Likewise, the scaffold-level cliff-density rankings provide a practical triage framework in which rugged scaffolds can be prioritized for denser analog exploration, whereas smooth scaffold families may provide comparatively lower-risk settings for physicochemical optimization.

The organization of the PAD4 SAR landscape also has implications for predictive modeling. A dataset dominated by smooth scaffold series but containing a small number of highly rugged regions represents a setting in which global performance metrics may obscure localized prediction failures. A model achieving strong overall RMSE may nevertheless perform poorly within the relatively small subset of rugged scaffold series that are most relevant for medicinal chemistry decision-making [27]. This interpretation is consistent with previous observations linking molecular-property landscape roughness to out-of-sample prediction error across diverse benchmark datasets [52], while activity-cliff distribution and chemical-space organization can further influence QSAR model performance [53]. PAD4-DB therefore provides an appropriate setting for evaluating whether model performance is preserved specifically within cliff-rich and scaffold-rugged regions rather than only at the global dataset level.

PAD4-DB therefore provides more than a curated bioactivity database. The annotated hub labels, MMP-supported transformations, scaffold-level ruggedness metrics, and activity-cliff annotations consolidated into a single per-compound resource table (Supplementary Table S5) establish a target-specific benchmark grounded in curated experimental bioactivity data for developing and evaluating cliff-aware machine-learning methods [54] and for assessing explainability approaches under chemically challenging conditions [30,55,56,57,58]. Future PAD4 QSAR and deep-learning models may benefit from incorporating uncertainty estimation or cliff-aware learning strategies rather than relying exclusively on global optimization objectives. Finally, the observed fingerprint sensitivity reinforces the need to anchor activity-cliff definitions in chemically meaningful shared substructures rather than a single fingerprint threshold: 64 of 94 severe cliff pairs fall below the Tanimoto threshold under ECFP6, yet 80% of these pairs (51/64) remain MMP- supported [59,60].

The principal contribution of PAD4-DB is not the discovery of new compounds—every compound in the resource originates from existing public repositories—but the systematic integration, normalization, and biological interpretation of these data into a unified medicinal chemistry resource. Compared with individual databases, PAD4-DB introduces consensus potency normalization, an explicit source-independence confidence framework, and comprehensive annotation of activity cliffs, cliff hubs, scaffold ruggedness, and matched molecular pair relationships. Cross-target benchmark resources such as MoleculeACE [27] have established that activity cliffs represent a widespread challenge for molecular machine learning; however, their broad cross-target scope necessarily limits the depth of target-specific curation that can be applied to any individual target. PAD4-DB complements these resources by providing substantially greater depth for a therapeutically important target that is attracting increasing interest in both inflammatory disease and oncology [2,3,4], thereby functioning simultaneously as a medicinal chemistry knowledge base and a rigorously curated benchmark for SAR analysis and predictive modeling.

Several limitations should be considered when interpreting these findings. First, consensus pIC_50_ values necessarily combine measurements originating from assay formats employing different calcium concentrations and experimental protocols, introducing systematic variability that reflects the well-established calcium sensitivity of PAD4 [17,20]. Second, the completeness of PAD4-DB remains constrained by the underlying public repositories; compounds such as GSK199, Pyroxamide, and PAD-PF1 remain absent because of upstream curation gaps, while patent-derived potency measurements are inherently more heterogeneous. Nevertheless, excluding all patent-exclusive compounds alters the severe activity-cliff count by only a single pair (94 to 93), indicating that the overall landscape is robust to this source of uncertainty. Third, the observed predominance of smooth SAR may partly reflect historical medicinal chemistry sampling rather than the intrinsic topology of PAD4 chemical space. Fourth, mechanistic interpretations of the identified cliff archetypes remain hypothetical because no structural validation was performed in the present study.

Fifth, the source-independence score is a heuristic metric based on known re-curation relationships among public repositories. Although rank-stability analyses support the robustness of the scoring scheme, the absolute threshold used to define non-redundant compounds should be interpreted as an operational convention rather than a universal measure of experimental independence.

Sixth, activity-cliff identification depends on the chosen fingerprint and similarity threshold. The ECFP6 sensitivity analysis shows that some ECFP4-defined cliffs are not robust at a larger radius, reinforcing the importance of MMP confirmation as a chemically interpretable structural-support layer.

Finally, although PAD4 represents an increasingly attractive therapeutic target, no PAD4 inhibitor has yet achieved regulatory approval. Accordingly, PAD4-DB should be interpreted as a computational and medicinal chemistry resource for organizing and analyzing preclinical bioactivity evidence rather than as evidence of clinical efficacy.

Overall, PAD4-DB demonstrates that careful data curation can generate substantially greater scientific value than aggregation alone. By integrating reproducible bioactivity standardization with quantitative characterization of scaffold arrangement, activity cliffs, and medicinal chemistry provenance, the resource establishes a foundation for optimizing future PAD4 inhibitors, supports the development of more reliable predictive models, and provides a transparent benchmark for computational methods applied to an increasingly vital therapeutic target in both autoimmune disease and oncology.

## 4. Materials and Methods

### 4.1. Data Sources and Target Identity

PAD4-DB was assembled for human PAD4 (UniProt Q9UM07) from 57 confirmatory and 11 literature-derived PubChem bioassays [35], 26 secondary PubChem bioassays, three high-throughput screening campaigns (AIDs 463073, 485272, and 488796), ChEMBL assay CHEMBL6111 [36,37], and the BindingDB record for UniProt Q9UM07 [38]. Target identity was audited per assay (93 PAD4-explicit assays, 2 PAD-family assays, and 2 ambiguous assays; all non-explicit assays contributed zero dose–response records). Species filtering retained *Homo sapiens* only. PubChem data were downloaded between 10 and 14 June 2026; ChEMBL data were downloaded on 14 June 2026; and BindingDB data were downloaded on 10 June 2026.

Two dual-layer assays (AIDs 1920046 and 2202442) were loaded from the confirmatory copy only, excluding 46 secondary rows. For the RFMS replicate assays AIDs 2202576 and 2202577, which shared 23.8% of PubChem substance identifiers (SIDs), AID 2202577 was preferred for the 55 overlapping compounds. AIDs 725596 and 725597 were confirmed to contain IC_50_ dose–response endpoints. Mixed Ki/Kd units in AID 1346144 were curated on a per-record basis.

### 4.2. Structure Standardization

Structures were processed with RDKit 2025.09.5 (Python 3.10.19; pandas 2.3.3; and numpy 2.2.5). BindingDB Daylight extended-SMILES annotations (|...|) were removed before parsing. Salts were stripped and the parent structure retained. Charge normalization was applied, but tautomer canonicalization beyond salt removal and charge normalization was not performed. Stereochemistry, by contrast, is preserved through standardization (isomeric SMILES; standard InChIKey with stereo layer), so distinct stereoisomers are treated as distinct compounds during deduplication; see Section 4.5. Deduplication used standard InChIKeys generated from the standardized structures. Of 341,282 ingested rows, 341,276 were standardized successfully (99.998%); the remaining six rows lacked structural input (NO_SMILES), and No parse or sanitization failures were observed among records containing structural input.

### 4.3. Activity Normalization

IC_50_-type concentration values were converted to pIC_50_ = −log_10_(IC_50_ [M]). Percent-inhibition HTS rows (*n* = 330,136) were intercepted before unit conversion to prevent spurious nanomolar conversion, increasing the proportion of records with valid endpoints from 89.8% to 99.0%. Records without numeric values (*n* = 3155) or with unconvertible units (*n* = 106; e.g., k_inact/K_I, k_on/k_off) were flagged and excluded from the dose–response potency set. Because PAD4 IC_50_ values can be sensitive to calcium concentration and assay format [17,20], consensus pIC_50_ values are reported together with source and assay-mechanism annotations rather than being treated as assay-independent measurements.

### 4.4. Replicate Aggregation, Deduplication, and Source-Independence Scoring

Replicates were consolidated by averaging pIC_50_ values, equivalent to geometric-mean aggregation of the underlying IC_50_ concentrations, within InChIKey × source × assay × endpoint groups. A total of 450 multi-replicate groups were identified. Compounds were then deduplicated to the InChIKey × source × endpoint level. Final per-compound consensus pIC_50_ values were obtained by averaging the curated source-level pIC_50_ values in log space, equivalent to geometric-mean aggregation of the corresponding IC_50_ concentrations. This process yielded 3093 structure-resolved dose–response inhibitors and 327,336 single-concentration HTS compounds. Overlap between the dose–response and HTS layers was assessed by exact InChIKey matching. HTS actives were defined as compounds with ≥50% inhibition at the reported screening concentration.

Each compound’s source combination was mapped to a source-independence score. Single-source compounds received a score of 1.0. The three-source BindingDB+ChEMBL+PubChem combination received the lowest score (0.3; *n* = 1366), reflecting known re-curation relationships among public repositories. Intermediate scores reflected the empirical re-curation hierarchy as follows: ChEMBL+PubChem received 0.7 (*n* = 23), BindingDB+ChEMBL received 0.6 (*n* = 167), and BindingDB+PubChem received 0.5 (*n* = 1199). Compounds with scores ≥0.6 were classified as non-redundant. Rank stability was confirmed by randomly perturbing the weights by ±20% (1000 draws; minimum Spearman rank correlation ρ = 0.93) and by comparing the result with an alternative inverse-source-count weighting scheme (ρ = 0.98). This hierarchy reflects documented cross-repository data flows as follows: BindingDB imports records directly from ChEMBL for entries with a well-defined protein target, and both BindingDB and ChEMBL contribute data to PubChem via PubChem BioAssays [35,36,38]. The extremes of the scale are well-grounded: single-source compounds carry no cross-import risk and receive the highest score, while triple-overlap compounds are exposed to all three documented pairwise import relationships simultaneously and receive the lowest score. The relative ordering among pairwise multi-source combinations (0.5, 0.6, and 0.7) is a heuristic refinement rather than a precisely calibrated measure of redundancy magnitude; the practical validity of the scoring scheme rests on its demonstrated empirical stability (Section 2.2) rather than on the precision of this ordering.

### 4.5. Scaffold, Fingerprint, and Cliff Analysis

Generic Bemis–Murcko scaffolds [31] were computed with RDKit 2025.09.5 [39]. Scaffold counts are RDKit-version-dependent and should be reproduced using the pinned environment provided with the code. Structural similarity was calculated using ECFP4 fingerprints (Morgan radius 2, 2048 bits) [61], consistent with established activity-cliff conventions [28,29]. All pairwise Tanimoto similarities were computed, and 358,416 pairs with Tanimoto ≥ 0.6 were retained for SAR landscape analysis.

Activity cliffs were defined using Tanimoto ≥ 0.8 and graded, mutually exclusive |ΔpIC_50_| bands as follows: severe, |ΔpIC_50_| ≥ 2.0; moderate, 1.5 ≤ |ΔpIC_50_| < 2.0; and broad, 1.0 ≤ |ΔpIC_50_| < 1.5. SALI scores were computed as |ΔpIC_50_|/(1 − Tanimoto) [62]. The SAR landscape was summarized using a structure–activity spectrum map, in which pairs were classified as non-descript, continuous, discontinuous, or severe based on their similarity and potency-difference profiles.

For network analysis, compounds were represented as nodes and severe cliff pairs as edges. Node degree was defined as the number of severe cliff edges incident to a compound, and the four highest-degree compounds were designated hub compounds.

Assay-mechanism classes (BAEE colorimetric, RFMS-confirmed enzymatic, fluorescence-polarization binding, and covalent) were assigned from assay metadata and manual curation. Electrophilic warheads were annotated using SMARTS patterns for chloroacetamidine, fluoroacetamidine, haloacetyl, and related reactive groups. t-SNE embeddings shown in Figure 1 were computed from ECFP4 fingerprints (perplexity = 30; 1000 iterations; fixed random seed) and were used for visualization only.

An ECFP6 (radius 3) sensitivity analysis identified 64 severe cliff pairs that fell below Tanimoto 0.80 at the larger radius; 51 of these 64 pairs (80%) remained MMP-supported. Hub dominance was 53.3% under ECFP6. As a further robustness check, we recomputed all pairwise similarities using chirality-aware ECFP4 fingerprints (useChirality = True). This reduced the severe cliff count from 94 to 88 pairs: 6 pairs fell below the Tanimoto ≥ 0.8 threshold once stereochemistry was incorporated, reflecting cases where the achiral fingerprint assigned Tanimoto = 1.0 to pairs of stereoisomers. Four of these six pairs involved hub compounds A1 (3 pairs) and A2 (1 pair); hubs B1 and B2 were unaffected. Re-ranking compounds by degree directly within the chirality-aware severe-cliff network recovers the same four compounds as the top four by degree, with the same greater-than-two-fold gap to the fifth-ranked compound (11 vs. 5). Hub concentration was correspondingly stable (53.2% achiral vs. 52.3% chirality-aware), indicating that the hub-organization finding is not an artifact of achiral fingerprinting. We report the achiral ECFP4 analysis as primary, consistent with standard practice [60], and note this sensitivity result as confirmatory.

### 4.6. Matched Molecular Pair Analysis

For severe cliff pairs, a shared chemical core was identified by seeded rdFMCS with element and bond-order matching, following matched-molecular-pair conventions [63,64]. Pairs were classified by the size of the changing fragment into single-atom, small-substituent, and medium-substituent changes, according to the fragment-size definitions provided in the Supplementary Information. A valid matched molecular pair required a connected shared core. Of the 94 severe cliff pairs, 80 (85.1%) yielded a valid connected MMP core. A separate transformation-typology analysis (Supplementary Table S4) used a different ring-complete MCS rule; because the two analyses used different criteria, their counts are not directly comparable and should not be summed.

### 4.7. Statistical Analysis

Comparisons of two independent distributions were performed using the Mann–Whitney U test, with rank-biserial correlation as the effect-size measure. Multi-group comparisons were performed using the Kruskal–Wallis test. Categorical enrichment was evaluated using Fisher’s exact test. Monotonic associations were assessed using Spearman rank correlation. Multiple-testing correction was performed using the Benjamini–Hochberg false discovery rate procedure where applicable.

Potency-permutation null models were used to assess the statistical significance of the observed activity-cliff landscape. In the unrestricted null model, pIC_50_ values were permuted across compounds in the Tanimoto ≥ 0.8 subgraph (2620 compounds), permuting at the compound level rather than the pair level, so that correlation induced by compounds appearing in multiple pairs is preserved identically in both the observed and null statistics, while the similarity structure was held fixed. In the scaffold-constrained null model, pIC_50_ values were permuted only within Murcko scaffold series. In the assay-constrained null model, pIC_50_ values were permuted within assay-mechanism classes. Each permutation test used 10,000 iterations with a fixed random seed. For each permutation, we recorded (i) the number of severe cliff pairs and (ii) the fraction of severe cliff edges incident to the four highest-degree compounds. Empirical *p* values were calculated as (k + 1)/(N + 1) [65], where k is the number of permutations yielding a statistic at least as extreme as the observed value and N = 10,000. Z-scores for hub enrichment were calculated as (observed − mean_null)/SD_null.

Robustness analyses were performed at |ΔpIC_50_| thresholds of 1.5, 2.0, and 2.5 (Supplementary Table S2). Automated pre-flight validation asserted the canonical dataset counts (3093 compounds; 94 severe cliff pairs; four hubs; and 1244 scaffolds) before figure generation. The MMP analysis was independently checked against the severe-tier subset, reproducing the 80/94 result reported in the Results; across all cliff tiers, the MMP pipeline identified 707 valid relationships and 24 unique cores.

## 5. Conclusions

PAD4-DB consolidates fragmented public bioactivity data into a curated SAR resource of 3093 PAD4 inhibitors and reveals a predominantly smooth landscape with severe activity cliffs concentrated around a small number of hub compounds. Of 12,071 cliff-candidate pairs, 94 met the severe activity-cliff criterion (80 MMP-supported; 14 fingerprint-only candidates), with 53.2% concentrated around four hub compounds, whereas 96.8% of multi-member scaffold series contained no severe cliff pairs. These hubs therefore identify localized regions of elevated SAR uncertainty that warrant targeted analog exploration and experimental validation. Importantly, 82.9% of the dataset was classified as pipeline-dependent under the heuristic source-independence framework, highlighting the need for provenance-aware data integration. Mechanism-class conflation between covalent and reversible inhibitors was not responsible for the observed cliff landscape: none of the 94 severe cliffs involved covalent-mechanism compounds, and excluding all 21 covalent compounds left the severe-cliff count unchanged. Overall, PAD4-DB provides a reproducible foundation for PAD4 inhibitor discovery and for evaluating prediction performance and uncertainty across chemically smooth and cliff-rich regions of PAD4 chemical space.

## Figures and Tables

**Table 1:** Source coverage and consensus potency.

Source	*n*	% of 3093	Mean pIC_50_	Median pIC_50_	SD
PubChem (confirmatory)	2821	91.2	6.62	6.85	0.90
BindingDB	2827	91.4	6.59	6.85	0.94
ChEMBL	1566	50.6	6.50	6.90	1.10
All three sources	1366	44.2	6.64	6.94	0.93

The first three rows report independent per-source coverage; percentages sum to >100% because compounds overlap across repositories. The “All three sources” row represents the triple-overlap intersection rather than an independent coverage category. pIC_50_ = −log_10_(IC_50_ [M]); values represent consensus across replicate measurements. Kruskal–Wallis H = 0.25, *p* = 0.88.

**Table 2:** Recovery of curated PAD4 reference inhibitors (*n* = 14).

Status	Compounds	Notes
Present, concordant (7)	Streptonigrin (5.602), Cl-amidine (5.219), F-amidine (4.571), GSK484 (7.049), TDFA (5.638), BMS-P5 (7.009), JBI-589 (6.000)	mean |ΔpIC_50_| < 0.15 vs. the literature
Present, not mapped (3)	o-F-amidine; Amodiaquine; BB-Cl-amidine	no primary IC_50_ endpoint (kinact/KI, HTS-only, or covalent kinetics)
Absent by design (3)	GSK199; Pyroxamide; PAD-PF1	not deposited in any source database
Correctly excluded (1)	AFM-30a	PAD2-selective; correctly absent from PAD4 dataset

Values in parentheses are consensus pIC_50_. GSK484 recovered as free base (InChIKey BDYDINKSILYBOL-WMZHIEFXSA-N) after salt stripping.

**Table 3:** Activity-cliff tiers.

Tier	|ΔpIC_50_| Band	Pairs	Compounds	% of Dataset	Median |ΔpIC_50_|	Max |ΔpIC_50_|
Severe	≥2.0	94	99	3.2	2.23	3.045
Moderate	1.5–<2.0	193	209	6.8	1.70	1.99
Broad	1.0–<1.5	580	539	17.4	1.17	1.50

All tiers require Tanimoto ≥ 0.8 (ECFP4, r = 2, 2048 bits). Compound counts are not additive (union of cliff-participating compounds = 654). % of dataset = compounds in tier/3093.

**Table 4:** Structural and network properties of the four severe-cliff hub compounds.

Hub ID	Archetype	InChIKey	Key Structural Motif	Consensus pIC_50_	Severe Cliff Pairs	Hub Contribution (%)	Scaffold Size (*n*)
A1	Class A (Series floor)	SMADULGDNOCLOP-GISFHXKWSA-N	Azaindole–benzimidazole core	5.39	15	16.0	174
A2	Class A (Series floor)	RAVBZQAQTVGKIV-XBPDSQQVSA-N	Azaindole–benzimidazole core	5.34	12	12.8	174
B1	Class B (Singleton attractor)	UDCDEKJNAMHBFH-HSZRJFAPSA-N	Cyclobutyl sulfonamide; free primary amine	4.30	12	12.8	1
B2	Class B (Singleton attractor)	DVCKJOQIVOGXEI-XMMPIXPASA-N	Cyclopentyl sulfonamide; free primary amine	4.30	11	11.7	1

The four hubs account for 50 of the 94 severe activity-cliff pairs (53.2%). Scaffold size (*n*) denotes the number of compounds sharing the same generic Bemis–Murcko framework. A1 and A2 share the azaindole–benzimidazole scaffold (S1, *n* = 174) but differ in their substituents. B1 and B2 are classified as distinct singleton scaffolds because the cyclobutyl and cyclopentyl ring systems are retained in the Bemis–Murcko framework, although they differ by only one methylene group (Tanimoto similarity = 0.975).

## Data Availability

All data and code supporting the findings of this study are openly available on Zenodo at https://doi.org/10.5281/zenodo.21815480 under a Creative Commons Attribution 4.0 International license. The associated GitHub repository is available at https://github.com/Nidhal212/PAD4-DB (accessed on 6 August 2026)).

## Supplementary Materials

The following supporting information can be downloaded at: https://www.mdpi.com/article/10.3390/ijms27188302/s1.

## References

[1] Wang Y., Li M., Stadler S., Correll S., Li P., Wang D., Hayama R., Leonelli L., Han H., Grigoryev S.A. (2009). Histone Hypercitrullination Mediates Chromatin Decondensation and Neutrophil Extracellular Trap Formation. J. Cell Biol..

[2] Yuzhalin A.E. (2019). Citrullination in Cancer. Cancer Res..

[3] Zhu D., Lu Y., Wang Y., Wang Y. (2022). PAD4 and Its Inhibitors in Cancer Progression and Prognosis. Pharmaceutics.

[4] Jia Y., Jia R., Taledaohan A., Wang Y., Wang Y. (2024). Structure-Activity Relationship of PAD4 Inhibitors and Their Role in Tumor Immunotherapy. Pharmaceutics.

[5] Wu C.-Y., Yang H.-Y., Lai J.-H. (2020). Anti-Citrullinated Protein Antibodies in Patients with Rheumatoid Arthritis: Biological Effects and Mechanisms of Immunopathogenesis. Int. J. Mol. Sci..

[6] Papayannopoulos V. (2018). Neutrophil Extracellular Traps in Immunity and Disease. Nat. Rev. Immunol..

[7] Spengler J., Lugonja B., Jimmy Ytterberg A., Zubarev R.A., Creese A.J., Pearson M.J., Grant M.M., Milward M., Lundberg K., Buckley C.D. (2015). Release of Active Peptidyl Arginine Deiminases by Neutrophils Can Explain Production of Extracellular Citrullinated Autoantigens in Rheumatoid Arthritis Synovial Fluid. Arthritis Rheumatol..

[8] Teijeira Á., Garasa S., Gato M., Alfaro C., Migueliz I., Cirella A., de Andrea C., Ochoa M.C., Otano I., Etxeberria I. (2020). CXCR1 and CXCR2 Chemokine Receptor Agonists Produced by Tumors Induce Neutrophil Extracellular Traps That Interfere with Immune Cytotoxicity. Immunity.

[9] Arita K., Hashimoto H., Shimizu T., Nakashima K., Yamada M., Sato M. (2004). Structural Basis for Ca2+-Induced Activation of Human PAD4. Nat. Struct. Mol. Biol..

[10] Bashir F., Awais H., Waseem A., Shahzad A., Babar Khan A., Ali S.A., Shafiq L., Ahmed Bhatti M. (2025). Structural and Mechanistic Insights into Peptidylarginine Deiminase (PAD2/PAD4) Mediated Citrullination and Therapeutic Targeting: A Review. Int. J. Biol. Macromol..

[11] Yang C., Dong Z.-Z., Zhang J., Teng D., Luo X., Li D., Zhou Y. (2021). Peptidylarginine Deiminases 4 as a Promising Target in Drug Discovery. Eur. J. Med. Chem..

[12] Luo Y., Arita K., Bhatia M., Knuckley B., Lee Y.-H., Stallcup M.R., Sato M., Thompson P.R. (2006). Inhibitors and Inactivators of Protein Arginine Deiminase 4: Functional and Structural Characterization. Biochemistry.

[13] Jones J.E., Slack J.L., Fang P., Zhang X., Subramanian V., Causey C.P., Coonrod S.A., Guo M., Thompson P.R. (2012). Synthesis and Screening of a Haloacetamidine Containing Library To Identify PAD4 Selective Inhibitors. ACS Chem. Biol..

[14] Knuckley B., Causey C.P., Jones J.E., Bhatia M., Dreyton C.J., Osborne T.C., Takahara H., Thompson P.R. (2010). Substrate Specificity and Kinetic Studies of PADs 1, 3, and 4 Identify Potent and Selective Inhibitors of Protein Arginine Deiminase 3. Biochemistry.

[15] Luo Y., Knuckley B., Lee Y.-H., Stallcup M.R., Thompson P.R. (2006). A Fluoroacetamidine-Based Inactivator of Protein Arginine Deiminase 4: Design, Synthesis, and in Vitro and in Vivo Evaluation. J. Am. Chem. Soc..

[16] Causey C.P., Jones J.E., Slack J.L., Kamei D., Jones L.E., Subramanian V., Knuckley B., Ebrahimi P., Chumanevich A.A., Luo Y. (2011). The Development of N-α-(2-Carboxyl)Benzoyl-N(5)-(2-Fluoro-1-Iminoethyl)-l-Ornithine Amide (o-F-Amidine) and N-α-(2-Carboxyl)Benzoyl-N(5)-(2-Chloro-1-Iminoethyl)-l-Ornithine Amide (o-Cl-Amidine) as Second Generation Protein Arginine Deiminase (PAD) Inhibitors. J. Med. Chem..

[17] Lewis H.D., Liddle J., Coote J.E., Atkinson S.J., Barker M.D., Bax B.D., Bicker K.L., Bingham R.P., Campbell M., Chen Y.H. (2015). Inhibition of PAD4 Activity Is Sufficient to Disrupt Mouse and Human NET Formation. Nat. Chem. Biol..

[18] Jia Y., Bahraminejad S., Duan X., Kong S., Taledaohan A., Jiang C., Ma D., Jiang J., Wang Y., Liu J. (2025). Discovery of Highly Potent Naphthalene/Quinoline-Based PAD Inhibitors: Structure-Activity Relationship, Selectivity, and Cytotoxicity. Eur. J. Med. Chem..

[19] Tjin C.C., Wissner R.F., Jamali H., Schepartz A., Ellman J.A. (2018). Synthesis and Biological Evaluation of an Indazole-Based Selective Protein Arginine Deiminase 4 (PAD4) Inhibitor. ACS Med. Chem. Lett..

[20] Dakin L.A., Xing L., Hall J., Ding W., Vajdos F.F., Pelker J.W., Ramsey S., Balbo P., Sahasrabudhe P.V., Banker M.E. (2025). Inhibiting Peptidylarginine Deiminases (PAD1-4) by Targeting a Ca2+ Dependent Allosteric Binding Site. Nat. Commun..

[21] Deng H., Lin C., Garcia-Gerique L., Fu S., Cruz Z., Bonner E.E., Rosenwasser M., Rajagopal S., Sadhu M.N., Gajendran C. (2022). A Novel Selective Inhibitor JBI-589 Targets PAD4-Mediated Neutrophil Migration to Suppress Tumor Progression. Cancer Res..

[22] Li M., Lin C., Deng H., Strnad J., Bernabei L., Vogl D.T., Burke J.J., Nefedova Y. (2020). A Novel Peptidylarginine Deiminase 4 (PAD4) Inhibitor BMS-P5 Blocks Formation of Neutrophil Extracellular Traps and Delays Progression of Multiple Myeloma. Mol. Cancer Ther..

[23] Gajendran C., Fukui S., Sadhu N.M., Zainuddin M., Rajagopal S., Gosu R., Gutch S., Fukui S., Sheehy C.E., Chu L. (2023). Alleviation of Arthritis through Prevention of Neutrophil Extracellular Traps by an Orally Available Inhibitor of Protein Arginine Deiminase 4. Sci. Rep..

[24] Aiken S.G., Grimes T., Munro S., Zarganes-Tzitzikas T., La Thangue N.B., Brennan P.E. (2025). A Patent Review of Peptidylarginine Deiminase 4 (PAD4) Inhibitors (2014–Present). Expert Opin. Ther. Pat..

[25] Zhao X., Gu C., Wang Y. (2020). PAD4 Selective Inhibitor TDFA Protects Lipopolysaccharide-Induced Acute Lung Injury by Modulating Nuclear P65 Localization in Epithelial Cells. Int. Immunopharmacol..

[26] Wang B., Su X., Zhang B., Pan S. (2023). GSK484, an Inhibitor of Peptidyl Arginine Deiminase 4, Increases the Radiosensitivity of Colorectal Cancer and Inhibits Neutrophil Extracellular Traps. J. Gene Med..

[27] van Tilborg D., Alenicheva A., Grisoni F. (2022). Exposing the Limitations of Molecular Machine Learning with Activity Cliffs. J. Chem. Inf. Model..

[28] Maggiora G.M. (2006). On Outliers and Activity CliffsWhy QSAR Often Disappoints. J. Chem. Inf. Model..

[29] Stumpfe D., Bajorath J. (2012). Exploring Activity Cliffs in Medicinal Chemistry. J. Med. Chem..

[30] Jiménez-Luna J., Skalic M., Weskamp N. (2022). Benchmarking Molecular Feature Attribution Methods with Activity Cliffs. J. Chem. Inf. Model..

[31] Bemis G.W., Murcko M.A. (1996). The Properties of Known Drugs. 1. Molecular Frameworks. J. Med. Chem..

[32] Langdon S.R., Brown N., Blagg J. (2011). Scaffold Diversity of Exemplified Medicinal Chemistry Space. J. Chem. Inf. Model..

[33] Bajorath J., Peltason L., Wawer M., Guha R., Lajiness M.S., Van Drie J.H. (2009). Navigating Structure–Activity Landscapes. Drug Discov. Today.

[34] Vogt M., Huang Y., Bajorath J. (2011). From Activity Cliffs to Activity Ridges: Informative Data Structures for SAR Analysis. J. Chem. Inf. Model..

[35] Kim S., Chen J., Cheng T., Gindulyte A., He J., He S., Li Q., Shoemaker B.A., Thiessen P.A., Yu B. (2025). PubChem 2025 Update. Nucleic Acids Res..

[36] Zdrazil B., Felix E., Hunter F., Manners E.J., Blackshaw J., Corbett S., de Veij M., Ioannidis H., Lopez D.M., Mosquera J.F. (2024). The ChEMBL Database in 2023: A Drug Discovery Platform Spanning Multiple Bioactivity Data Types and Time Periods. Nucleic Acids Res..

[37] Papadatos G., Gaulton A., Hersey A., Overington J.P. (2015). Activity, Assay and Target Data Curation and Quality in the ChEMBL Database. J. Comput. Aided. Mol. Des..

[38] Liu T., Hwang L., Burley S.K., Nitsche C.I., Southan C., Walters W.P., Gilson M.K. (2025). BindingDB in 2024: A FAIR Knowledgebase of Protein-Small Molecule Binding Data. Nucleic Acids Res..

[39] Landrum G., Tosco P., Kelley B., Rodriguez R. (2025). RDKit: Open-Source Cheminformatics. Zenodo.

[40] Lex A., Gehlenborg N., Strobelt H., Vuillemot R., Pfister H. (2014). UpSet: Visualization of Intersecting Sets. IEEE Trans. Vis. Comput. Graph..

[41] Copeland R.A. (2013). Evaluation of Enzyme Inhibitors in Drug Discovery.

[42] Jasial S., Hu Y., Bajorath J. (2016). Assessing the Growth of Bioactive Compounds and Scaffolds over Time: Implications for Lead Discovery and Scaffold Hopping. J. Chem. Inf. Model..

[43] Hu H., Bajorath J. (2020). Simplified Activity Cliff Network Representations with High Interpretability and Immediate Access to SAR Information. J. Comput. Aided. Mol. Des..

[44] Hu H., Bajorath J. (2020). Introducing a New Category of Activity Cliffs Combining Different Compound Similarity Criteria. RSC Med. Chem..

[45] Brambilla M., Zanichelli A., Cancila V., Colombo M.P., Chiodoni C., Sangaletti S. (2025). Neutrophil Extracellular Traps in Cancer: Immune Modulation, Therapy Resistance, and the Dilemma of Targeting. Cell Death Dis..

[46] Hu W., Lee S.M.L., Bazhin A.V., Guba M., Werner J., Nieß H. (2023). Neutrophil Extracellular Traps Facilitate Cancer Metastasis: Cellular Mechanisms and Therapeutic Strategies. J. Cancer Res. Clin. Oncol..

[47] Albrengues J., Shields M.A., Ng D., Park C.G., Ambrico A., Poindexter M.E., Upadhyay P., Uyeminami D.L., Pommier A., Küttner V. (2018). Neutrophil Extracellular Traps Produced during Inflammation Awaken Dormant Cancer Cells in Mice. Science.

[48] Cools-Lartigue J., Spicer J., McDonald B., Gowing S., Chow S., Giannias B., Bourdeau F., Kubes P., Ferri L. (2013). Neutrophil Extracellular Traps Sequester Circulating Tumor Cells and Promote Metastasis. J. Clin. Investig..

[49] Jia Y., Bahraminejad S., Jiang C., Taledaohan A., Ma D., Jiang J., Wang Y., Liu J. (2025). New Structural Scaffolds to Enhance the Metabolic Stability of Arginine-Derived PAD4 Inhibitors. Results Chem..

[50] Zou P., Su X., Tian W., Xie X., Yang K. (2026). Peptidylarginine Deiminase 4-Mediated Citrullination in Human Disease: Molecular Mechanisms and Therapeutic Targeting. Front. Pharmacol..

[51] Griffen E., Leach A.G., Robb G.R., Warner D.J. (2011). Matched Molecular Pairs as a Medicinal Chemistry Tool. J. Med. Chem..

[52] Aldeghi M., Graff D.E., Frey N., Morrone J.A., Pyzer-Knapp E.O., Jordan K.E., Coley C.W. (2022). Roughness of Molecular Property Landscapes and Its Impact on Modellability. J. Chem. Inf. Model..

[53] López-Pérez K., Miranda-Quintana R.A. (2025). Extended Activity Cliffs-Driven Approaches on Data Splitting for the Study of Bioactivity Machine Learning Predictions. Mol. Inform..

[54] Chen X., Yu D., Zhao L., Liu F. (2025). ACES-GNN: Can Graph Neural Network Learn to Explain Activity Cliffs?. Digit. Discov..

[55] Szostek T., Szulczyk D. (2026). From Obstacle to Design Advantage: Activity Cliff Aware Modeling for Small-Molecule Drug Discovery. Drug Discov. Today.

[56] Abe R., Miyao T., Bajorath J. (2026). Accurate Prediction of Activity Cliff Compounds Based on Bioactivity Profiles Depends on Assay Nearest Neighbor Relationships. J. Cheminform..

[57] Zhang Z., Zhao B., Xie A., Bian Y., Zhou S. (2023). Activity Cliff Prediction: Dataset and Benchmark (ACNet). arXiv.

[58] Chen H., Vogt M., Bajorath J. (2022). DeepAC—Conditional Transformer-Based Chemical Language Model for the Prediction of Activity Cliffs Formed by Bioactive Compounds. Digit. Discov..

[59] Peltason L., Iyer P., Bajorath J. (2010). Rationalizing Three-Dimensional Activity Landscapes and the Influence of Molecular Representations on Landscape Topology and the Formation of Activity Cliffs. J. Chem. Inf. Model..

[60] Rottach F., Schieferdecker S., Eickhoff C. (2025). The Topology of Molecular Representations and Its Influence on Machine Learning Performance. J. Cheminform..

[61] Rogers D., Hahn M. (2010). Extended-Connectivity Fingerprints. J. Chem. Inf. Model..

[62] Guha R., Van Drie J.H. (2008). Assessing How Well a Modeling Protocol Captures a Structure-Activity Landscape. J. Chem. Inf. Model..

[63] Hussain J., Rea C. (2010). Computationally Efficient Algorithm to Identify Matched Molecular Pairs (MMPs) in Large Data Sets. J. Chem. Inf. Model..

[64] Hu X., Hu Y., Vogt M., Stumpfe D., Bajorath J. (2012). MMP-Cliffs: Systematic Identification of Activity Cliffs on the Basis of Matched Molecular Pairs. J. Chem. Inf. Model..

[65] Phipson B., Smyth G.K. (2010). Permutation P-Values Should Never Be Zero: Calculating Exact P-Values When Permutations Are Randomly Drawn. Stat. Appl. Genet. Mol. Biol..

