# Supplementary material for: PAD4-DB: A Curated Structure–Activity Resource Reveals Hub-Organized Activity Cliffs and Scaffold-Dependent SAR Ruggedness in PAD4 Inhibitors
Source: Int J Mol Sci. 2026 Sep 17;27(18):8302. doi: 10.3390/ijms27188302 (PMC13607315; doi:10.3390/ijms27188302)
Supplement: Supplementary file 1 [file ijms-27-08302-s001.zip › Supplementary_Figures.pdf]

Supplementary Figures

PAD4-DB: a curated structure–activity resource reveals hub-organized activity cliffs and scaffold-dependent SAR ruggedness in PAD4 inhibitors.

This file contains Supplementary Figures S1–S5. Supplementary Tables S1–S4 are provided as separate files. Additional quality-control and analysis tables are available in the project GitHub repository and Zenodo deposition.

Figure S1. Scaffold-level severe-cliff density

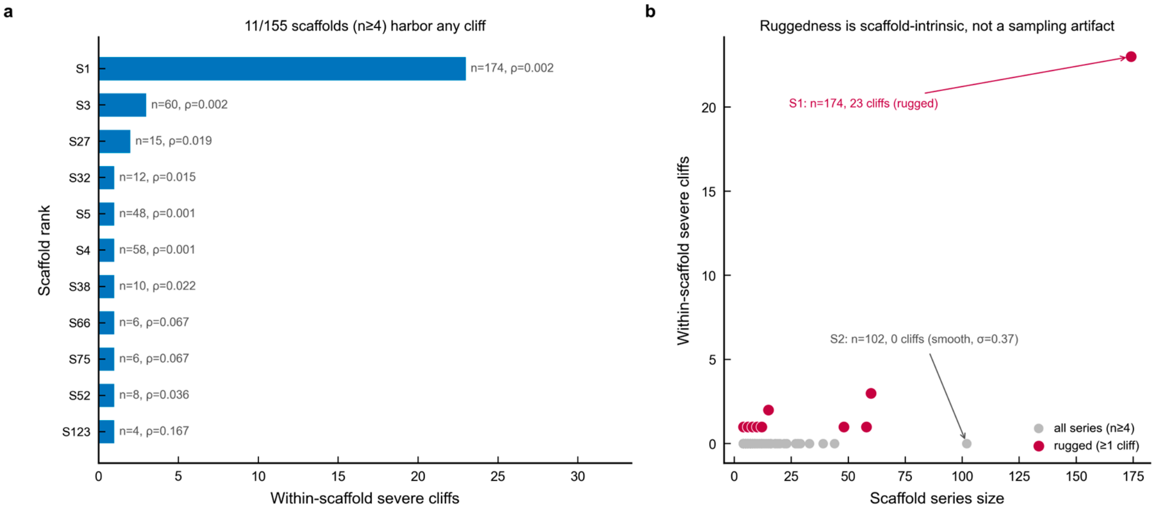

Distribution of severe activity cliffs across Bemis–Murcko scaffold series. Most multi-member series contain no severe cliff pairs. The largest series, S1, contains 23 severe cliff pairs.

Figure S2. Assay-mechanism enrichment of severe cliffs

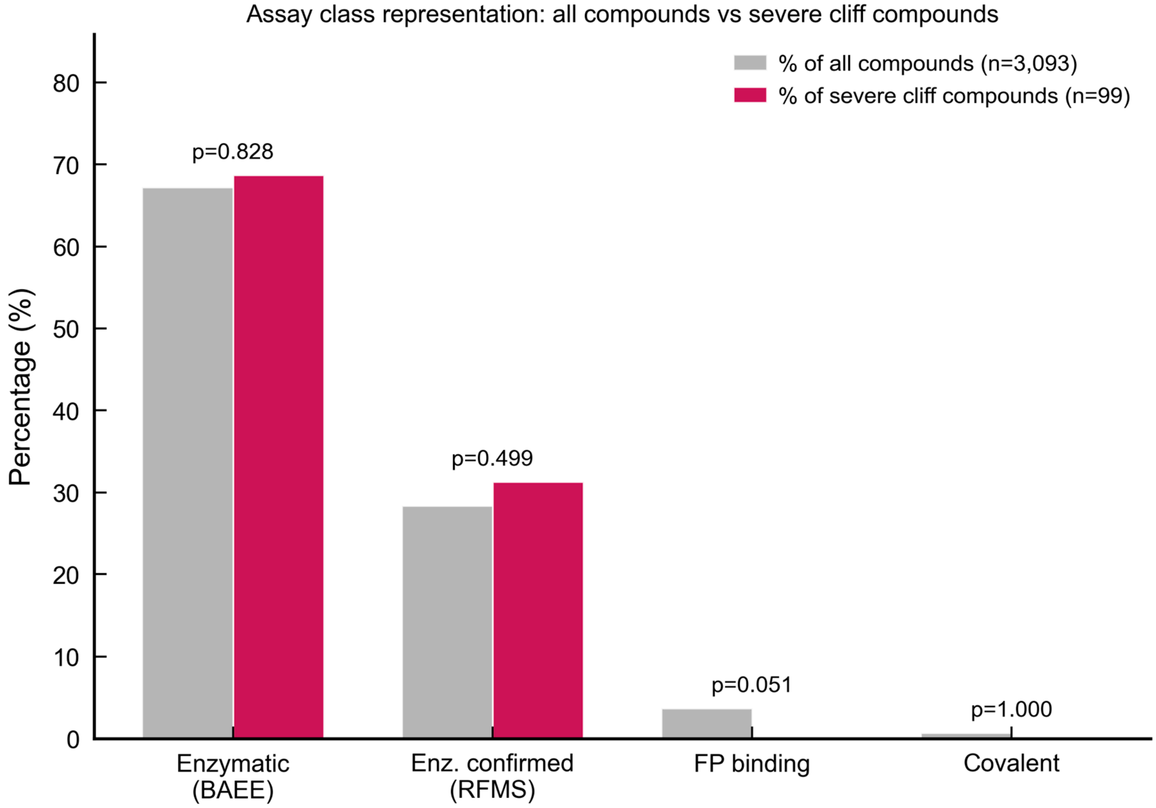

Severe-cliff compounds are not significantly enriched in any assay-mechanism class (Fisher's exact test; all P > 0.05), supporting the assay independence of the identified cliff landscape.

Figure S3. Structure–activity spectrum map

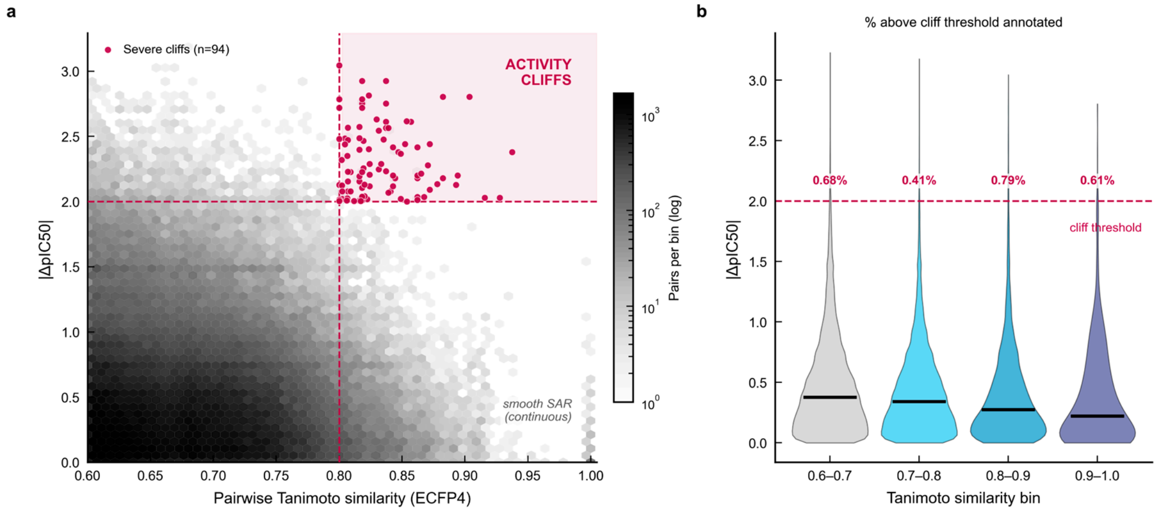

Structure–activity spectrum (SAS) map of 358,416 structurally related compound pairs (Tanimoto ≥ 0.6). The landscape is dominated by non-descript and continuous pairs, with severe activity cliffs representing 0.026% of pairs.

Figure S4. Scaffold ruggedness panels

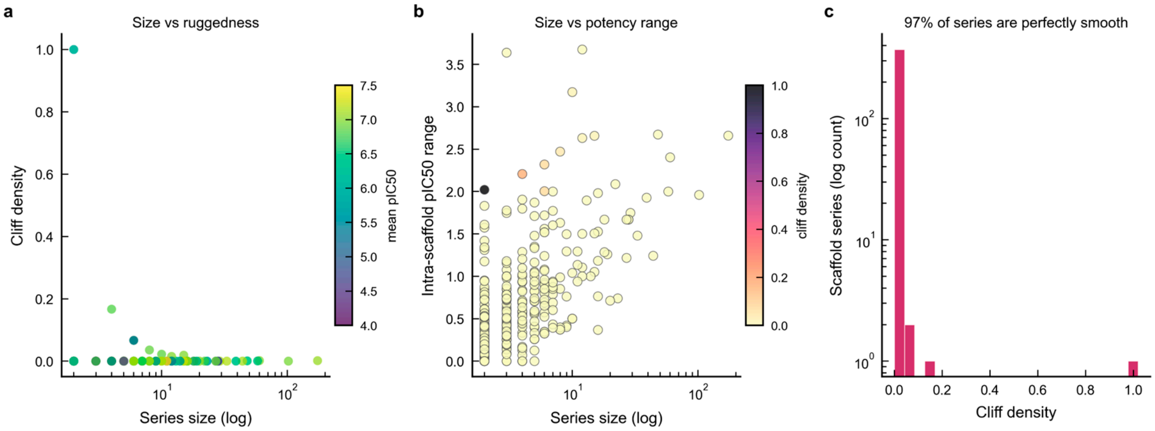

Additional panels illustrating heterogeneous intra-scaffold potency spread. Ruggedness is rare and localized: only 12 of 375 multi-member scaffold series contain any within-scaffold severe activity cliff.

Figure S5. Null-model comparisons

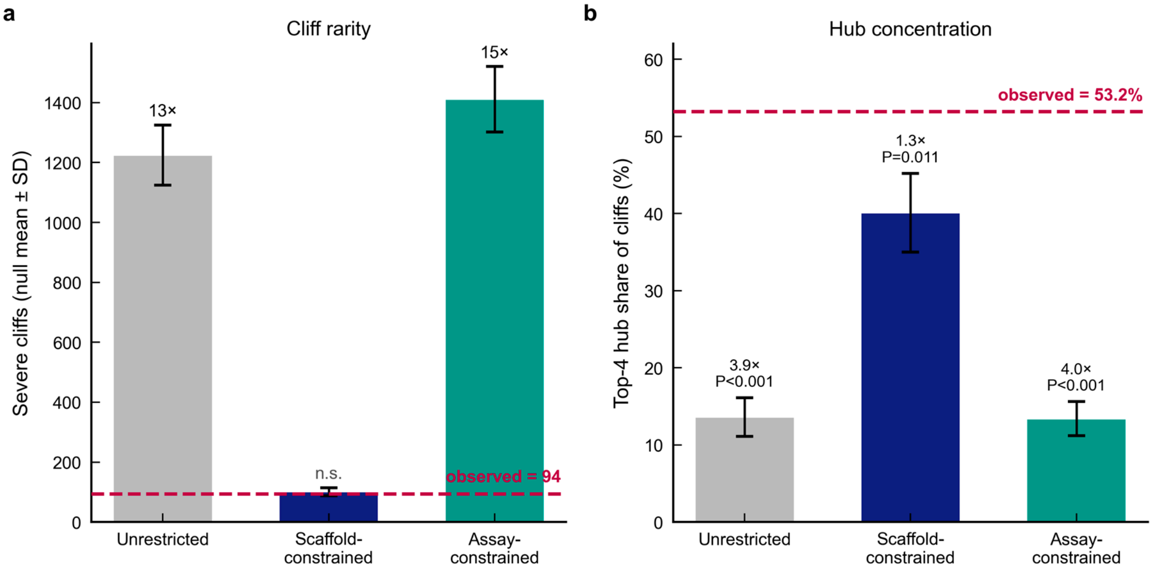

Severe-cliff counts and hub concentration under unrestricted, scaffold-constrained, and assay-constrained permutation models. Hub concentration remains significant under scaffold- and assay-constrained null models.
